# Progress in THz Rectifier Technology: Research and Perspectives

**DOI:** 10.3390/nano12142479

**Published:** 2022-07-19

**Authors:** Rocco Citroni, Franco Di Paolo, Patrizia Livreri

**Affiliations:** 1 Department of Electronic Engineering, University of Rome Tor Vergata, 00133 Roma, Italy; franco.di.paolo@uniroma2.it; 2 Department of Engineering, University of Palermo, 90128 Palermo, Italy; patrizia.livreri@unipa.it

**Keywords:** energy harvesting, rectifying antenna, quantum tunneling, ballistic transport, quasi-ballistic transport

## Abstract

Schottky diode (SD) has seen great improvements in the past few decades and, for many THz applications, it is the most useful device. However, the use and recycling of forms of energy such as solar energy and the infrared thermal radiation that the Earth continuously emits represent one of the most relevant and critical issues for this diode, which is unable to rectify signals above 5 THz. The goal is to develop highly efficient diodes capable of converting radiation from IR spectra to visible ones in direct current (DC). A set of performance criteria is investigated to select some of the most prominent materials required for developing innovative types of electrodes, but also a wide variety of insulator layers is required for the rectification process, which can affect the performance of the device. The current rectifying devices are here reviewed according to the defined performance criteria. The main aim of this review is to provide a wide overview of recent research progress, specific issues, performance, and future directions in THz rectifier technology based on quantum mechanical tunneling and asymmetric structure.

## 1. Introduction

The energy that reaches the Earth through solar radiation (1000 W/m^2^ at sea level in clear weather conditions), extended from ultraviolet (5%) to infrared (IR) (55%) through the visible region (40%), can be collected and converted to direct current (DC). For the IR region, the most important component is the MID-IR wavelength re-emitted from the surface of the Earth from 8 to 14 μm with maximum emissivity at 10.6 μm (28.3 THz). Today, solar panels based on the photovoltaic effect represent the most common harvester capable of capturing only visible radiation and converting it into useful DC stored for later use. This solution, combined with rechargeable batteries, makes any device self-powered with a virtually infinite lifetime. Despite the sun providing enough energy to power the entire planet, there is a problem. Photovoltaic cells are not efficient enough to convert solar radiation into electricity. This is due to the ability to harvest only energy from the visible spectrum (400 to 750 nm) of the sun, whereas the MID-IR wavelength remains untapped by current solar cells. The conversion efficiency of silicon (Si)-based solar cells is limited to around 22% and in any case, cannot exceed the theoretical limit imposed by Shockley–Queisser (S&Q) [1,2]. The infrared thermal radiation that the Earth continuously emits into the cold outer space is around 10^17^ W. Technologies able to better harness the radiation around us are needed. If considering the radiation (from IR to optical) as an electromagnetic (EM) wave, and not as a corpuscle (photons), an alternative solution, theoretically more efficient than solar cell, can be introduced. Direct conversion of EM radiation into DC through a rectification process represents a possible solution to directly harvest solar energy as a complementary technology to photovoltaic. This concept is possible by introducing the rectenna, contraction of RECTifying antENNA [3,4]. This device is very attractive at MID-IR and visible frequencies and can have a significant impact in many areas. For terrestrial applications, solar radiation is null at night or in the presence of fog and is greatly reduced in humid areas. For the space applications, the success of any interplanetary exploration passes through correct generation of electricity in situ obtained by solar panels. However, they are not capable of producing electricity during night hours and dust storms. The IR radiation emitted by the Earth persists night and day; therefore, rectennas tuned in MID-IR could be implemented with rectennas tuned in the visible range to power small devices night and day.

**Figure 1:**
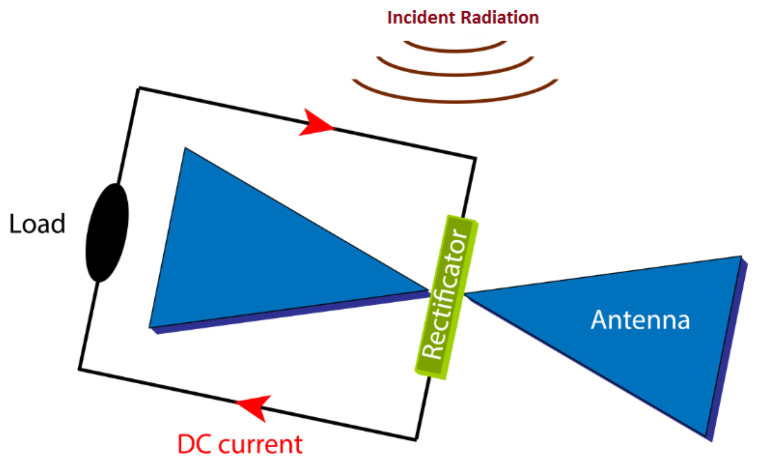
Architecture of a typical THz rectenna.

Rectennas at THz frequencies are considered potentially more efficient than solar cells that exceed the S&Q limit. Currently, the use of an antenna integrated with a rectifying diode has only been successfully demonstrated for microwave and radio frequency energy harvesting [4], because as the operation frequency increases (at infrared and visible frequencies), the conversion efficiency decreases. Cutting-edge efficiency of MID-IR rectennas even using 2D materials indicates values well below 1% [5,6,7,8,9,10,11,12,13,14,15]. In the visible range with the use of carbon nanotubes (CNTs) it was possible to obtain conversion efficiency of up to ≈10^−6^%. This low efficiency value is due to several impediments such as fabrication challenges but also impedance mismatching between antenna (hundreds of ohms) and diode (TΩ) [16,17,18]. The architecture of the whole system with the related details is shown in Figure 1. It consists of three blocks: an antenna at the nanoscale, a diode optimized to rectify the high frequency signal, and a load. These components are commonly associated with other components not visible in Figure 1 that will be discussed in the text.

In the first block, when the size of the antenna coincides with the wavelength of the incident EM wave, an energy transfer between them occurs. In this condition, called resonance condition, the collective oscillations of conduction electrons propagate at the metal-dielectric interface. This wave is referred to as surface plasmon polaritons (SPPs) and is localized at the interface between dielectric and metal with opposite sign permittivity. SPPs have the characteristic of decaying very rapidly as the distance from the metal surface increases. The movement of the SPPs induced by the intensity of the electric field (*E*) of the radiation of the incident EM wave generates an alternating current (AC) on the surface of the antenna. This flow is conveyed to the feed point of the antenna where the electric field (*E*) intensity obtained is one hundred times greater than the incident radiation. The second block is constituted of an ultra-high-speed diode. The diode must be optimized to rectify higher frequency. By assuming appropriate impedance matching between the equivalent antenna resistance and the equivalent diode resistance, needed to reach good conversion efficiency, when SPPs flow reaches the end of the antenna, it is captured by the diode positioned in the antenna feed point, obtaining a current flow in a single direction. At THz regimes, the diode must have a very short response time, i.e., it should have a lower RC time constant (10^−15^ s) than the AC cycle period. In principle, devices based on the electron tunnel respond to THz radiation with no losses. Furthermore, the open circuit voltage of the single antenna is about a few tens of picovolt and this might be insufficient for the rectification process to be efficient. The only way to overcome this problem is to design a diode that turns on at a voltage equal to the open circuit voltage of the antenna. The output voltage of the rectifier is not stable and is affected by ripples. To overcome this issue, a low pass filter (LPF) not visible in Figure 1 is introduced at the output of the circuit to produce a more stable and regular DC voltage. A DC-DC converter is also required to adapt the LPF output voltage levels to the level required by a storage device, usually a rechargeable battery not visible in Figure 1 that stores the excess energy for later use. Finally, the third block represents a load powered by a constant signal (DC). The load could be a resistor, a capacitor, an inductor, or a combination of all these elements [1,2,19,20]. The diode at the THz frequency plays a crucial role in improving the conversion efficiency of EM waves to DC. For energy harvesting (EH) applications, only the zero bias rectification process is considered of interest. Despite recent advancements, there are still a number of unresolved technologic challenges at these frequencies. 

This review aims to provide a wide overview of recent research progress, specific issues, performance, and future directions on THz rectifier technology based on quantum tunneling (single insulator and multi-insulator diodes) and ballistic or quasi-ballistic transport.

This paper is organized as follows: Section 2 discusses the status of planar diode technology (single and multi-insulator), including operation, performance criteria, and applications from IR to optical response. Section 3 describes the status of geometric diode technology (up to 28.3 THz), including operation, performance criteria, and applications. Finally, Section 4 provides perspectives and conclusions. 

### Rectification Efficiency (η)

Currently, the efficiency obtained for this technology is less than 1%, well below the efficiency of PV solar cells (about 22%). The energy conversion efficiency of a nanorectenna essentially depends on two elements, the antenna and the rectifier element. The efficiency of the antenna reflects its ability to concentrate the radiation and guide it in the desired direction. The efficiency of the rectifier is related to its ability to rectify the generated alternate current into direct current. However, there are numerous loss mechanisms for each stage of the energy conversion process. Therefore, the efficiency of the antenna depends on the combined efficiency of several factors such as the ability to couple the incident electromagnetic radiation and the dissipative losses within the antenna structure. Other factors such as the matching of the impedance to the nanorectifier and the coupling efficiency of the nanorectifier to the load also affect the efficiency. The overall efficiency *η* for a nanorectenna can be written as:

(1) $$\eta =\eta_{a}\eta_{S}\eta_{c}\eta_{q}$$

where

- $\eta_{a}$ is the efficiency of the coupling between the incident EM radiation and the antenna.
- $\eta_{S}$ is the efficiency given by the ratio between the energy transmitted to the nanodiode and the energy collected by the nanoantenna.
- $\eta_{c}$ is the coupling efficiency between the nanoantenna and the rectifier.
- $\eta_{q}$ is the efficiency given by the ratio between the rectified power and the power received by the nanodiode.

The radiation pattern and the bandwidth of the nanoantenna, the material conduction property, a poor AC-to-DC rectification at THz frequencies, and the impedance mismatch between the antenna element (hundreds of ohm) and the diode used for rectification (several kilo ohms) affect the overall efficiency of the nanorectenna-based energy harvester. 

## 2. THz Diode Technology: General Information, FOMs, Open Questions, State of the Art, and Perspectives

This section introduces a detailed discussion of THz diode technology, in particular, general information, figures of merit (FOMs), open questions, state of the art, and perspectives on metal-insulator-metal (MIM), metal-multi-insulator-metal diodes (MI^n^M), and geometric diode (GD). The aforementioned diodes are governed by two different mechanisms: quantum-tunneling effect; ballistic and quasi-ballistic transport. Although there have been continuous improvements for these diodes, they are not yet optimized for THz frequencies. Therefore, the greatest efforts of the scientific community are aimed at design and manufacturing.

### 2.1. THz Diode Technology: General Information on Metal Insulator Metal (MIM) Diode

The metal-insulator-metal (MIM) diode is an ultra-fast switching device able to operate at THz frequency range, thanks to the quantum tunneling effect. The MIM diode consists of a thin dielectric layer within two similar or dissimilar metal electrodes. The purpose of an MIM is to receive a very high frequency AC signal and to rectify it into DC signal. The time it takes for the electron to cross the forbidden band gap of the insulator layer must be less than the inversion time of the AC. This is possible if an insulator barrier is less than 5 nm, so that the tunneling effect can be maintained at the switching frequency THz.

**Figure 2:**
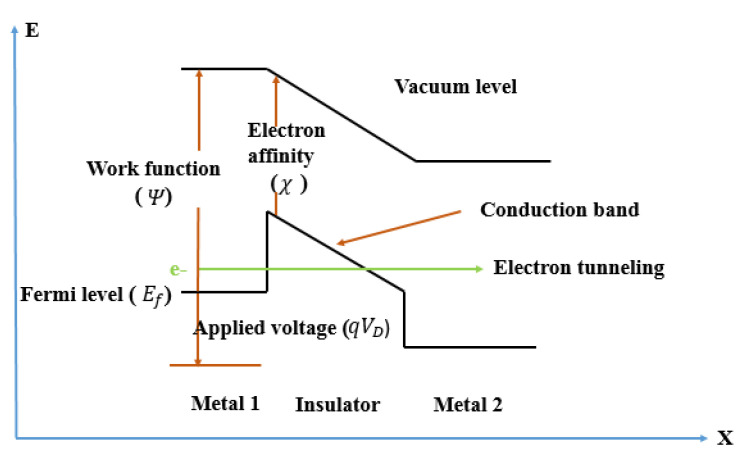
Energy-band profile of an MIM tunnel diode.

Currently, this type of diode can rectify signals up to a frequency of 343 THz [21], useful for reaching the visible regime. Figure 2 shows an energy-band diagram of an MIM tunnel barrier. The barrier height *φ* at the metal-insulator (M-I) interface is determined by the difference between the work function Ψ and the electron affinity *χ*; the width of the barrier is defined by the insulator thickness; and Δ*φ* represents the difference of heights of the barrier between the upper and the lower metal contacts [7,22]. 

**Figure 3:**
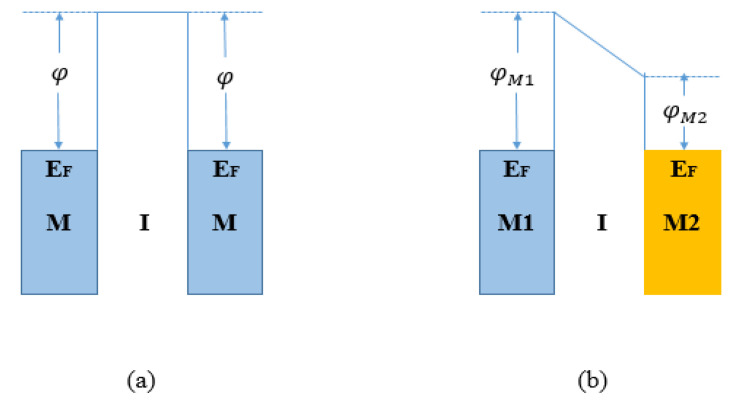
Band structure for (**a**) symmetric and (**b**) asymmetric MIM diodes under zero bias condition.

The conduction mechanism of an MIM diode is observed under three different bias conditions (zero bias, reverse bias, and forward bias). In Figure 3, the band structures under zero bias condition for symmetric and asymmetric MIM diodes are shown. In zero bias condition, when two metals are in contact, a net flow of electrons is observed from both sides, until the equilibrium condition is reached with an alignment of their Fermi levels (*E_F_*) and a potential barrier is formed between the metal contacts. A diode is symmetric if similar electrodes create equal energy barrier heights at each M-I interface due to the same work function, as shown in Figure 3a [7,23,24,25]. In this condition, the shape of the energy band diagram of the junction of the metal-insulator-metal with no bias voltage is rectangular. Consequently, a larger potential barrier at an M-I interface reduces the tunneling probability. This type of diode at zero bias condition is not able to rectify an electromagnetic wave and an external bias is required to have a nonzero current density. 

In Figure 3b, dissimilar electrodes presenting different work functions create different energy barrier heights at each M-I interface. 

As a result, an asymmetric tunneling current is generated and a trapezoidal barrier replaces the rectangular barrier so that the tunneling occurs more easily and a net flow of current through the device will be possible under zero bias conditions.

**Figure 4:**
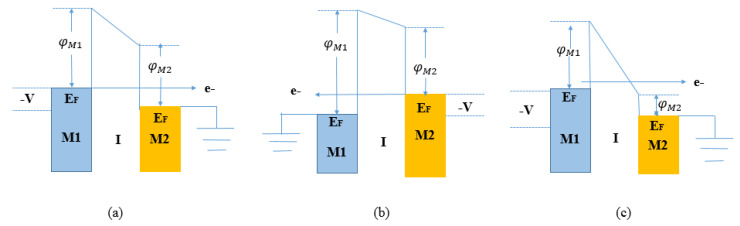
Band structure for asymmetric MIM diode: (**a**) forward bias, (**b**) reverse bias, and (**c**) more forward bias.

If the electron affinity of the insulator is close to one of the metal work function values, the band bending results in a triangular shape and this increases the tunneling current density through the barrier. Figure 4 shows the band structure of an asymmetric MIM diode for (a) forward bias, (b) reverse bias, and (c) more forward bias. 

In Figure 4a, the negatively biased metal contact M1 has a higher work function than the second grounded metal contact M2. Consequently, an increase in *E_F_* is observed in the M1 side with a decrease in the barrier height at M1-I. A net flow of electron tunnels through the entire dielectric thickness and direct tunneling (DT) represents the dominant conduction mechanism. This condition is known as the forward bias condition. In Figure 4c, under more forward bias conditions, the bias voltage *V* further increases, so the trapezoidal barrier becomes triangular and a net flow of electrons will cross a reduced tunneling distance. In this case, the Fowler–Nordheim tunneling (FNT) represents the dominant conduction mechanism. Finally, in Figure 4b, under a reverse bias condition, the negatively biased metal contact M2 has a higher work function than the first grounded metal contact M1. Consequently, the band diagram shows a small shift in its shape owing to the rise of the EF of M2. The barrier height at the M2-I interface decreases so electrons flow through the entire dielectric thickness. This results in reduced tunneling probability and, accordingly, in lower current density. In light of the considerations above discussed, efficient rectification requires a large forward-to-reverse current ratio.

### 2.2. THz Diode Technology: I-V Characteristics and Figures of Merit (FOM) for MIM Diode

The parameters that indicate the performance of an MIM diode are referred to as figures of merit (FOM) (asymmetry, nonlinearity, and responsivity). These parameters, combined with current density, turn-on voltage (TOV), and zero-bias resistance (ZBR), are influenced by several factors such as the combination of metal and insulator, the thickness of the insulation, and the barrier height between the M-I interface. 

#### 2.2.1. Current Density

Simmons et al. derived Equation (2) for the tunneling condition of electrons through a barrier of any arbitrary shape [26]:

(2) $$J=\frac{1.1\;q^{2}}{4\pi h}\frac{1}{\varphi }{\left(\frac{V+\Delta \varphi }{d}\right)}^{2}\times \exp\left(\frac{-23\pi \sqrt{qm}}{6h}\varphi_{}^{\frac{3}{2}}\left(\frac{d}{V+\Delta \varphi }\right)\right)$$

where *J* represents the tunneling-current density, *q* represents the electric charge, *h* represents Planck’s constant, *V* represents the applied bias, *φ* is the barrier height in the metal-insulator interface (M-I), Δ*φ* is the difference in barrier heights between top and bottom metal contacts, *m* represents the effective electron mass, and *d* represents the tunnel barrier thickness. As *d* decreases, the probability of electrons passing through the barrier layers increases, thus maintaining the tunnel effect. 

#### 2.2.2. Transmission Probability D

If an insulator layer is less than 3 nm thick, an electron may tunnel through it and appear almost instantly on the other side. This condition, indicated as transmission probability *D*, is given by Equation (3) [7,23,27,28,29]:

(3) $$D=e^{\left(-2d\sqrt{\frac{2m(V-E)}{\hbar^{2}}}\right)}$$

where *V* is the barrier height, *E* is the energy of electron, and *d* is the thickness of the insulator. 

The probability of an electron to pass through the insulator bandgap depends on *V* and *d*; in fact, as *d* and *V* increase, *D* decreases. 

#### 2.2.3. Turn-On Voltage (TOV)

The turn-on voltage (TOV) represents a positive voltage applied to the diode to “turn it on” and conduct current in the forward direction. In general, the TOV of these diodes has to be equal to the open circuit voltage of the antenna, which is in the range of a few picovolts. This small value could be insufficient for the rectification process. An increase in the TOV is possible if the insulator layer is thinner [7,23,27,28,29].

#### 2.2.4. Zero-Bias Resistance (ZBR)

Differential resistance *r_D_* given by Equation (4) and zero-bias resistance (ZBR) given by Equation (5) influence the behavior of the diode. The first resistance is obtained as a differentiating current with respect to the applied voltage [7,23,27,28,29]:

(4) $$r_{D}=\frac{1}{I^{\prime }},\;\mathrm{where}\;I^{\prime }=\frac{dI}{dV}$$

Its value increases exponentially with *d*; therefore, to obtain low resistance, barrier heights and insulator thickness must be small. 

The second resistance ZBR, evaluated at zero-bias voltage, is obtained:

(5) $$R^{ZB}={\left(\frac{dV}{dI}\right)}_{V=0}$$

To ensure maximum transfer of energy captured by the antenna towards the load, the ZBR value must coincide with that of the antenna’s resistance. This condition only occurs if the barrier heights on both sides of the metals are low and the insulator layer is very thin (typically less than 3 nm in the case of diodes with a single insulator layer).

#### 2.2.5. Asymmetry (*Asym*)

To achieve rectification, an ideal diode must show an asymmetrical I-V curve. This parameter is referred to as asymmetry. The asymmetry is defined as the absolute ratio of the forward current *I_F_* to the reverse current *I_R_*.

(6) $$Asym\;\;(V)=\left|\frac{I_{F}(V)}{I_{R}(V)}\right|;\mathrm{Asymmetry}\;>\;1$$

A value of 1 obtained by considering a diode of equal metals on both sides of the insulator layer indicates full symmetry and hence no rectification. If the diode consists of different metals on both sides of the insulator layer, the structure is asymmetric and the value is greater than 1. According to Equation (7), to achieve asymmetry and high current density, metals with a high difference in work function, good conductivity, and a large barrier height are desirable:

(7) $$Asym=10^{3.3137\left(\Psi_{M2}-\Psi_{M1}\right)}$$

where $\Psi_{M2}$ represents the work function of the top metal (M2) and $\Psi_{M1}$ represents the work function of the bottom metal (M1). The asymmetry also depends on the insulator thickness; as it increases, the asymmetry increases [7,23,27,28,29].

#### 2.2.6. Nonlinearity (*NL*)

All diodes must be fabricated with strong nonlinearity to achieve a large response, especially for applications such as EH where it is desirable to operate with no application of external DC bias. The nonlinearity in Equation (8) represents the ratio between the differential conductance and the conductance [7,23,27,28,29].

(8) $$NL\;(V)=\frac{\frac{dI}{dV}(V)}{\frac{I(V)}{V}};\;\mathrm{Nonlinearity}\;>\;3$$

Equation (8) represents the deviation from a linear resistor. The introduction of dissimilar metal electrodes and thick tunneling layers allows the obtaining of high nonlinearity. Furthermore, this parameter increases with the insulator thickness and the low barrier height.

#### 2.2.7. Responsivity (*S*)

In Equation (9), the responsivity, also called sensitivity (*S*), is expressed as the ratio of the second derivative to the first derivative of the I-V curve at a specific bias voltage [7,23,27,28,29].

(9) $$S=\frac{I^{\prime \prime }}{I^{\prime }},\;\mathrm{with}\;S\;>\;7V^{-1}$$

where $I\prime \prime =\frac{dI^{2}}{d^{2}V}$ and $I\prime =\frac{dI}{dV}$.

This parameter represents the rectified signal measurement vs. the input power. The greater the numeric value of responsivity, the greater the rectification ability of the diode. To achieve great responsivity, a large curvature in the I-V graph is required. It is useful to introduce another parameter, referred to as zero-bias responsivity, which represents the measure of the rectified signal as a function of the input power at zero-bias voltage. If this requirement is not met, the diode requires an external signal to generate a response that prevents the rectenna from operating as an energy harvester. In general, a trade-off is observed. In fact, as the barrier height increases, the responsivity increases too, and as a consequence, higher resistance is also observed.

### 2.3. THz Diode Technology: Open Questions

The first planar diodes for THz applications were made in the 1990s [4]. However, some questions as to the overall efficiency still remain open. At present, the overall efficiency is well below the theoretical predictions of around 0.001%, preventing the commercial development of this technology [7]. This value can improve if only some conditions are met. While there are currently fewer impediments in the fabrication process of the antenna, at optical and IR spectra, unfortunately, it is not the same for diodes, which have to meet many requirements as the operating frequency increases. Nanoscale antennas and related structures exhibit potential benefits in terms of production, fine-tuning, and high confinement of the electric field (*E*) intensity in the gap [30,31,32,33]. However, losses in noble metals negatively affect the antenna efficiency and consequently the overall efficiency [34,35]. Therefore, a careful choice of materials is required. Moreover, complete matching between the antenna and the rectifier to transfer maximum power to the load is mandatory. In addition, the open circuit voltage and the available power obtained by the rectenna are approximately a few tens of microvolt and a few picowatts, respectively. It is clear that an arrangement in an array is necessary. The practical realization of this array would require further studies to avoid parasitic interactions among them. Regarding the rectifier diode, the main issues not yet resolved at these frequencies concern the design and model, fabrication, characterization, and integration to the antenna. The requirement for highly precise fabrication processes, such as deposition of smooth metal electrodes, ultrathin oxide layer, and patterning nanoscale devices, are some other open questions. Finally, the reverse-bias leakage, lower RC time constant, and coupling efficiency problems limit its applicability. The single parameters that influence the behavior of the diode are shown in Table 1 and are explained in detail.

Asymmetry is one of the parameters that can be engineered. A high difference in the work function (Δ*φ*) between the metal electrodes involves high asymmetry and nonlinearity; moreover, these two parameters increase linearly by increasing the layer thickness. The thin dielectric layer and the low barrier height allow high transmission probability which increases exponentially with the decrease of the insulator thickness. To minimize TOV and maximize asymmetry and nonlinearity, the electron affinity of the insulator should be close to one of the metal work function values to produce a low barrier height. The cut-off frequency of the diode is governed by the RC (resistance-capacitance) time constant. The capacitance has to be kept as small as possible to increase the operating frequency of the MIM diode. The contact area and the thickness of the insulator layer mainly affect the capacitance. By increasing the thickness *d* of the insulator layer or reducing the contact area, it is possible to minimize the capacitance. However, as the thickness of the dielectric layer increases, the nonlinearity of I-V characteristics increases and the probability of tunneling decreases. In addition, the large contact area and short length induce negative effects on the resistance. 

The contact area effect is proportional to the capacitance and is inversely proportional to the resistance. In other words, there is a trade-off so that the resistance and the capacitance cannot decrease simultaneously. Coupling efficiency is obtained by taking into account some considerations during the MIM design. The conductivity and resistivity of the metals of the electrodes should have a high value to achieve high current density and a negligible value, respectively. Moreover, a great difference in work function is required to guarantee asymmetry. For insulators, great electron-affinity values that are quite equal to the metal work function ones are preferred since they produce a small band-offset at the interface needed to guarantee nonlinear characteristics. Furthermore, the dielectric constant should have a low value in order to cope with the reduced tunneling resistance. The narrow bandgap allows a sharp turn-on voltage. Finally, to increase the asymmetric barrier of the tunnel, it is possible to use stacking of insulators (MI^n^M devices), which have different band gaps and electron affinities (*χ*) [24,28,29,36,37,38,39].

### 2.4. THz Diode Technology: State of the Art of Metal Insulator Metal (MIM) Diode

The introduction of graphene as one of the possible electrodes is justified by virtues such as the band gap tunability, the very high electrical conductivity, and the thin-film fabrication process on a rigid or flexible substrate. In recent years, diodes based on graphene referred to as metal-insulator-graphene (MIG) have been realized. With the addition of boron nitride insulator (H-BN), it is possible to reduce the resistance of MIG. The work function of graphene is adjustable through the application of an electrical field, chemical doping, metal deposition, or plasma treatment. For EH application, the work functions of graphene must be adjusted using the chemical doping method [14,40,41]. The state of the art and the latest results for MIG and MIM are presented as follows. A study conducted by [23] shows an Al/AlO_x_/Gr printed on a Si/SiO_2_ substrate able to rectify up to 30 THz. The results obtained in terms of FOMs indicate *Asym* of 2500 at bias voltage of 1 V, an *NL* of 3.8, and zero-bias resistance (ZBR) of 600 Ω with a high current density *J_ON_* up to 1 A/cm^2^ for a 1 V bias. A Gr/TiO_2_/Ti printed on a Si/SiO_2_ substrate with 1 μm thermal SiO_2_ is shown in [42]. The results obtained in terms of FOMs indicate an *Asym* up to 520, a maximum *NL* up to 15, a maximum *S* up to 26 V^−1^, and a current density *J_ON_* of 7.5 A/cm^2^ for 1 V bias. For RF power detection, *S* is 2.8 V^−1^ at 2.4 GHz and 1.1 V^−1^ at 49.4 GHz. A Ti/TiO_2_/Bilayer Gr printed on a Si/SiO_2_ substrate with 300 nm thermal SiO_2_ is presented in [43]. The results obtained in terms of FOMs indicate a high *Asym* up to 9000, a maximum *NL* up to 8, a maximum *S* up to 10 V^−1^, and a current density *J_ON_* of 0.1 A/cm^2^ for 1 V bias. The best result was obtained in [44], by considering a graphene/hexagonal boron nitride (h-BN)/graphene heterostructure (Gr-h-BN-Gr). This structure, printed on a Si/SiO_2_ substrate with 90 nm thermal SiO_2_, consists of graphene as contacts and hexagonal boron nitride (h-BN) as insulator with a thickness less than 6 nm. The results obtained in terms of FOMs indicate an *Asym* of 1000, an *NL* of 40, a zero-bias *S* of 2.75 V^−1^, a peak *S* of 12 V^−1^, and a current density *J_ON_* of 0.02 A/cm^2^ for a bias of 1 V. The latest studies on MIG configurations and related FOMs discussed in this subsection are summarized in Table 2. In [8], the authors present an antenna integrated with a rectifier for harvesting infrared energy. The antenna consists of a resonant bowtie antenna that has been optimized to resonate at operating frequency of 28.3 THz and to produce highly enhanced localized fields inside the gap. The authors fabricated a Cu (100 nm)-CuO-Au (100 nm) (0.0045 μm^2^) MIM diode with a very small contact area (67 nm × 67 nm) and a very small oxide thickness (0.7 nm) to decrease the values of its capacitance and resistance (a relatively low zero-bias resistance of 500 Ω), respectively. 

**Figure 5:**
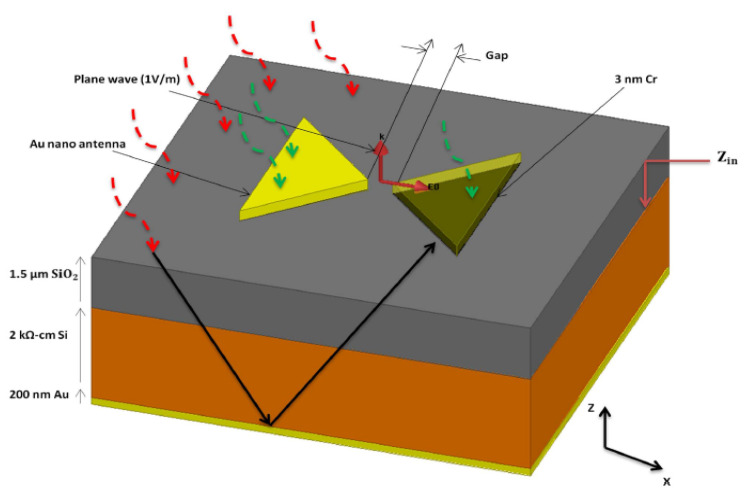
THz antenna integrated with a Cu (100 nm)-CuO-Au (100 nm) (0.0045 μm^2^) rectifier (rectenna) for harvesting infrared energy. Reprinted with permission from ref. [8]. Copyright 2014 Scientific Reports.

**Figure 6:**
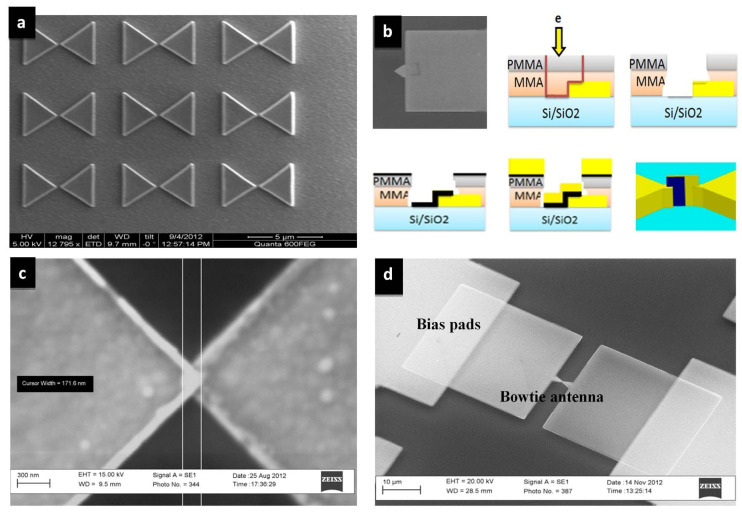
Fabrication of nanoantennas and the rectenna device. (**a**) SEM image of the nanoantenna array fabricated using EBL. (**b**) Overlap fabrication process: (i) first antenna arm, (ii) second arm exposure using EBL, (iii) removing the exposed resist using a mixture of MIBK and IPA developer with ratio of 153, (iv) deposition of 0.7 nm of oxide using atomic layer deposition (ALD), (v) second arm sputtering, (vi) complete device after the liftoff process using acetone. (**c**) SEM image of the fabricated overlap. (**d**) SEM image of the antenna-integrated diode. Reprinted with permission from ref. [8]. Copyright 2014 Scientific Reports.

The MIM diode, developed using electron beam lithography (EBL), guarantees the low resistance requirement and the THz signal rectification with no external electrical source. The structure of the THz antenna integrated to the diode is shown in Figure 5. It is composed of a four-layer stack-up. The first is a chromium thin-film layer (3 nm) for adhesion between the gold nanoantenna and the substrate. The second is a silicon dioxide layer (1.5 μm), deposited by plasma-enhanced chemical vapor deposition (PECVD), to increase the THz radiation transmission into the silicon substrate. The third is a thick silicon layer (375 μm) with high resistivity (2 KΩ·cm) to reduce substrate losses. The fourth is a thin gold back reflector layer (200 nm) to enhance coupling to the antenna and the substrate. The fabrication of the nanorectenna is shown in Figure 6. The diode is composed of two different metals, gold and copper, with a copper oxide in between as an insulator.

Gold (the lower antenna arm) and copper (the upper antenna arm) have a work function of 5.1 eV and 4.7 eV, respectively. The simulation results are carried out by considering a normally incident plane wave (*z*-axis) with an electric field intensity of 1 V/m and linear polarization parallel to the antenna axis (*x*-axis). Bias pads (20 μm × 32 μm) are connected to the antenna. The results indicate maximum responsivity *S* (V^−1^) equal to 6, zero-bias responsivity equal to 4, and zero-bias resistance equal to 505 Ω. 

The fabrication and characterization of Ti-TiO_2_-Al and Ti-TiO_2_-Pt tunnel diodes are given in [45]. In both cases, the thickness of the oxide is approximately 9 nm. The dioxide is formed by means of two different techniques, native oxide growth and plasma oxidation. In the first case, a silicon substrate is used for the MIM. The thickness of the titanium lower metal layer is of 70 nm. The thickness of the aluminum upper metal is of 150 nm. In the second case, the thickness of the titanium is of 80 nm. Measurements indicate for Ti-TiO_2_-Al (21,287 μm^2^ area) an *S* of 18 V^−1^, at DC voltage of 0.09 V, an *NL* of 6.5 at 0.33 V, and a current density *J_ON_* of 10^−1^ A/cm^2^ at 1 V. The results for Ti-TiO_2_-Pt indicate an *S* of 15 V*^−^*^1^ at DC voltage of 0.495 V, an *NL* of 15 at 0.5 V, and a current density *J_ON_* of 10^0^ A/cm^2^ at 1 V. In [46], the authors present fabrication and characterization of eight Nb/Nb_2_O_5_-based MIM diodes. The thickness of the Nb_2_O_5_ layer is 15 nm, whereas the thickness of the Nb layer is around 100 nm. For all eight MIM combination diodes, the highest reported rectification speed (∼150 THz) has been obtained. Samples were fabricated on p-type Si wafers. Measurement indicates promising results for Nb/Nb_2_O_5_/Cu, Nb/Nb_2_O_5_/Ag, Nb/Nb_2_O_5_/Au, and Nb/Nb_2_O_5_/Pt. In particular, Nb/Nb_2_O_5_/Pt exhibits an *Asym*, an *NL*, and an *S* of 1500, 4, and 20 V^−1^ at 0.5 V, respectively, whereas Nb/Nb_2_O_5_/Cu, Nb/Nb_2_O_5_/Ag, and Nb/Nb_2_O_5_/Au exhibit an *Asym*, an *NL*, and an *S* of 1500, 8, and 20 V^−1^ at 150 mV, respectively. The design, fabrication, and characterization of an asymmetric diode Au/Al_2_O_3_/Pt operating up to 28.3 THz printed on 1.5 µm of Si substrate are given in [47]. The Au/Al_2_O_3_/Pt diode exhibits a highly nonlinear I-V characteristic due to which high zero-bias responsivity of 10 V^−1^ is achieved with an on current of 6 × 10^−7^ A around 1 V. In [48], the authors present high-frequency Ni-NiO-Ag metal-insulator-metal tunnel diodes fabricated via anodic aluminum oxide (AAO) templates. With a contact area of 3.1 × 10^−4^ µm^2^ and an insulator thickness (NiO) of 6 nm, the results obtained indicate a current of 150 µA around 1.0 V, *Asym* of 5 around 1.0 V, *NL* of 3.0 around 0.5 V, zero-bias responsivity *S* (V^−1^) of 5.8, and maximum responsivity *S* (V^−1^) of 8.5 around 0.1 V. These results indicate great potential for high-frequency applications. In [49], the authors present metal-insulator-metal diodes based on alkyltrichlorosilane self-assembled monolayers (SAMs) with different alkyl chain lengths. The insulator SAM is in between two metal contacts, Pt (5.65 eV) and Ti (4.33 eV). The electronic properties of the MIM diodes can be tuned by controlling the alkyl chain length of the SAMs. Alkyltrichlorosilane (SiCl_3_–(CH_2_)_n−1_–CH_3_) SAMs are ultrathin high-quality dielectric films with a well-controlled structure. The diodes were fabricated on 2-inch Si wafers with a 300 nm thermally grown SiO_2_ layer. Standard photolithography was used to pattern the metal contacts. The alkyltrichlorosilane SAM is sandwiched between two metal contacts, Pt and Ti. The overlapping region between the Ti and Pt contacts determines the effective area of the diode, which is 100 µm^2^. The zero-bias responsivity S for the diode based on 1.20 nm, 1.34 nm, 1.57 nm, and 2.23 nm films was found to be 4.1, 4.6, 4.7, and 8.0 V^−1^, respectively. In addition, the zero-bias dynamic resistance *R_d_* for the diodes based on 1.20 nm, 1.34 nm, 1.57 nm, and 2.23 nm films was found to be 32 kΩ, 71 kΩ, 464 kΩ, and 5 GΩ, respectively, as the thickness increases. In addition, parameters such as asymmetry, nonlinearity, and responsivity indicate that the diode based on 2.23 nm has the highest *Asym*, strongest *NL*, and highest *S*, the values of which are 117.8, 6.8, and 20.8 V^−1^, respectively. The only negative aspect for this asymmetric diode is a high *R_d_* value that involves a potential mismatch between radiative element and diode rectifier. In [29], the authors present metal–insulator–metal diodes. The best results were obtained for two diodes, Nb/Nb_2_O_5_/Pt and Nb/TiO_2_/Pt, with 13 nm of insulator layer. These two types of diodes show a low TOV of 100 mV and an operating frequency up to 30 THz. At a voltage bias of 0.5 V, the Nb/Nb_2_O_5_/Pt MIM structure exhibits *NL* and *Asym* values of ∼3.8 and ∼130, respectively, while the Nb/TiO_2_ /Pt MIM structure exhibits *NL* and *Asym* values of ∼3.5 and ∼80, respectively. In [27], the authors fabricated several metal-insulator-metal diodes based on the Nb/Nb_2_O_5_/X material system. The symbol X represents the second electrode (M2) realized by several materials combinations: Nb, Ag, Cu, Ni, Au, and Pt. The thickness of the Nb_2_O_5_ was typically 15 nm. The top contact metals were deposited and patterned using a lift-off procedure on the Si/Nb/Nb_2_O_5_ samples to create devices with a 6400 μm^2^ active area. Six different top contact metals were combined: Ag, Cu, Ni, Au, and Pt to Nb/Nb_2_O_5_; the highest FOMs at 0.5 V are obtained for Nb/Nb_2_O_5_ (15 nm)/Ni (300 K) and Nb/Nb_2_O_5_ (15 nm)/Au (300 K). 

*Asym*, *NL*, and *S* are 396.5, 7.1, 8.5 V^−1^ for Nb/Nb_2_O_5_(15 nm)/Ni (300 K), and 1430.8, 8.0, 7.0 V^−1^, respectively, for Nb/Nb_2_O_5_ (15 nm)/Au (300 K). In [50], the authors present a new metal-insulator-metal capacitor based on SrTiO_3_/Al_2_O_3_/SrTiO_3_ for RF applications. The MIM capacitors were fabricated on a 5 μm SiO_2_ deposited on silicon wafers. The thinner Al_2_O_3_ induces a higher capacitance density at the price of degrading leakage current while the thicker Al_2_O_3_ would cause the opposite effects. Tantalum nitride (TaN) was deposited and patterned to form the top electrodes. The electrodes of all samples were deposited using 500 nm thick Al for good ohmic contacts. The results indicate a very high capacitance density of 19.13 fF/μm^2^ due to the perovskite SrTiO_3_ (STO) with a very high dielectric constant of 145. 

This MIM capacitor also displays a quadratic voltage coefficient of 610 ppm/V^2^ and a good leakage current of 5 × 10^−9^ A/cm^2^ at 2 V, which is attributed to the inserted Al_2_O_3_. This combination of materials makes this diode a promising candidate for higher frequencies. In [51], the authors present a fabrication and characterization of high-sensitivity copper-copper oxide-copper (Cu-CuO-Cu) metal-insulator-metal tunnel junctions. The MIM diode was fabricated on 300 nm of silicon dioxide (SiO_2_) placed on silicon (Si) substrates. Electrodes patterning was conducted by electron beam lithography. Cu electrodes were shown as 100 nm thick and 2 nm of CuO was deposited using RF sputtering. In this study, there were Cu-CuO-Cu symmetrical MIM tunnel junctions with 2 × 2 μm^2^ of contact area; the results indicate maximum responsivity of 4.497 V^−1^ at a bias voltage of 153 mV and zero-bias dynamic resistance *R_d_* of 180 KΩ. In [52], the authors present quantum-tunneling metal-insulator-metal diodes made by rapid atmospheric pressure chemical vapor deposition. The authors realized and characterized an asymmetric diode Pt/Al_2_O_3_/Al printed on 500 nm of SiO_2_ deposited on Si. The diode consists of Al_2_O_3_ with thickness of 6 nm sandwiched between Pt/Al with thickness of 100 nm. Materials and deposition are indicated in [52]. The results are obtained by considering two different methods, atmospheric pressure chemical vapor deposition (AP-CVD) and plasma-enhanced atomic layer deposition (PEALD) techniques. With the first method, Pt–Al_2_O_3_-Al shows an *Asym* of 110 for 1.5 V, an *NL* of 6 for 1.4 V, an *S* of 9 V^−1^ for 1.2 V, and current voltage (I-V) responses of 1 × 10^−10^ A with a voltage bias of 2 V, TOV of 1.4 V, and zero-bias dynamic resistance of *R_d_* = 5 × 10^12^ Ω. With the second method, Pt–Al_2_O_3_-Al shows an *Asym* of 30 for 2 V, an *NL* of 30 for 1.8 V, an *S* of 22 V^−1^ for 1.8 V, and current voltage (I-V) responses of 1 × 10^−11^ A with a voltage bias of 2 V, TOV of 1.75 V, with zero-bias dynamic resistance *R_d_* of 7 × 10^12^ Ω. In [53], the authors present a model, design, and fabrication of antenna (bowtie)-coupled metal-insulator-metal diodes for IR sensing. In detail, an asymmetric diode Al-Al_2_O_3_-Au designed to rectify up to 60 THz is given. Metal-insulator-metal diodes were fabricated on 100 nm SiO_2_ placed on Si substrates. Three layers of device were patterned by using the electron beam lithography (EBL) system. The bottom layer was 495 K polymethyl methacrylate (PMMA) and the top layer was 950 K PMMA. Bilayer resist was used in lithography. The first electrode made of aluminum is 65 nm thick whereas different thicknesses of Al_2_O_3_ were deposited in order to investigate the thickness dependence of the characteristics of the devices. In the second electrode, 5 nm of chromium (Cr) followed by 65 nm thick gold (Au) were deposited. 

The results obtained indicate an *S* of 14.46 V^−1^ for 600 mV, a density current voltage (J–V) response of 4.0 μA/cm^2^ for a voltage bias of 800 mV, a current of 12 nA, and zero-bias dynamic resistance *R_d_* of 100 kΩ for 0.6 V voltage bias. In [19], the authors present a model, design, fabrication, and characterization of an asymmetric Al-Al_2_O_3_-Cr MIM diode able to rectify up to 28.3 THz. For the fabrication of MIM diode, the aluminum (work function of 4.28 eV) is used for the preparation of the bottom electrode, and aluminum dioxide Al_2_O_3_ (having electron affinity of 1.25 eV) is used as a barrier layer after plasma oxidation of aluminum. For the top electrode, chromium is used, having sufficiently higher work function (4.5 eV). The substrate on which the MIM diode is fabricated is a microscopic glass slide, which is optically flat and smooth on both sides. The whole structure is printed on 1.5 µm of SiO_2_ deposited on Si. The diode is realized by considering a thin-film insulator layer Al_2_O_3_ with thickness of 3 nm sandwiched between two different metals Al/Cr with thickness of 100 nm. The results obtained indicate a maximum current density (J-V) of 2 × 10^−4^ A/cm^2^ at 0.8 V and *NL* of 3.1 at 0.8 V. The latest studies on MIMs discussed in this subsection are summarized in Table 3.

### 2.5. THz Diode Technology: General Information on Metal Multi-Insulator Metal (MI^n^M) Diode

**Figure 7:**
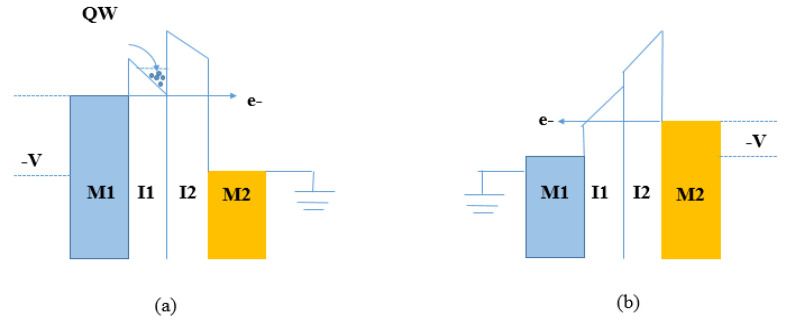
Resonant tunneling: (**a**) forward bias, (**b**) reverse bias.

It is very difficult to combine requirements such as FOMs and low resistance in a single MIM diode. High value of nonlinearity and high asymmetry cannot be reached simultaneously. Asymmetry is achieved with metals presenting large differences in their band gaps (energy levels increase), whereas high nonlinearity is obtained with thicker barriers and larger barrier heights. This combination leads to high diode resistance. In general, the addition of another insulator layer allows precise control of asymmetry and nonlinearity. Therefore, to obtain low resistance, a possible solution is multiple insulator layers of a few nanometers thickness sandwiched between two similar or dissimilar metal electrodes. This device is referred to as MI^n^M, where n represents the number of ultrathin insulator layers. The use of multi-insulator layers with dissimilar affinity and band gap leads to different band offsets obtained at metal-interface (M-I), interface-interface (I-I), and interface-metal (I-M). The tunneling current can be easily engineered by modifying the band offsets through biasing. The introduction of two insulator layers (*n* = 2) with very thin thickness, referred to as metal-insulator-insulator-metal (MIIM), introduces three different barrier height values, localized among metal-insulator (M-I), insulator-insulator (I-I), and insulator-metal (I-M) interfaces. The barrier height value at each interface governs parameters such as tunneling efficiency and FOMs. This approach allows one to create an additional asymmetry in the tunnel barrier and to achieve precise control over nonlinearity. The asymmetry can further be increased with different metal electrode combinations [7,23,54,55,56,57,58]. However, metals with similar work functions can also be used as long as the electron affinity of both insulators is different, so as to keep the resistance low. Although there have been continuous improvements, this type of diode is not yet optimized for THz frequencies. In fact, there are several open issues. By inserting three or more insulator layers between two metal contacts, this leads to an increase in the tunneling resistance, reducing the current. In addition, an external bias voltage is needed to move electrons out of the quantum well (QW) created by the two insulator layers. Consequently, this rectifying diode is less attractive for EH applications. In light of what has been said, the mechanisms that govern this device are based on resonant tunneling and step tunneling. Both these mechanisms are explained in detail as follows. Figure 7 shows the schematic energy band diagram of the resonant tunneling MIIM diode in forward and reverse conditions. Resonant tunneling occurs when a triangular quantum well (QW) (an area where electrons may exist) is created between the two insulators with different band gaps (one with a large band gap and another one with a smaller band gap). An external bias voltage controls the flow of electrons.

In the forward bias condition in Figure 7a, a negative bias voltage is applied to the electrode with higher work function (to the left metal contact M1), whereas the electrode (M2) with lower work function is grounded. The Fermi level of M1 increases and when it reaches the lowest energy level of the well, electrons from M1 begin to tunnel through the insulator barrier using this path.

**Figure 8:**
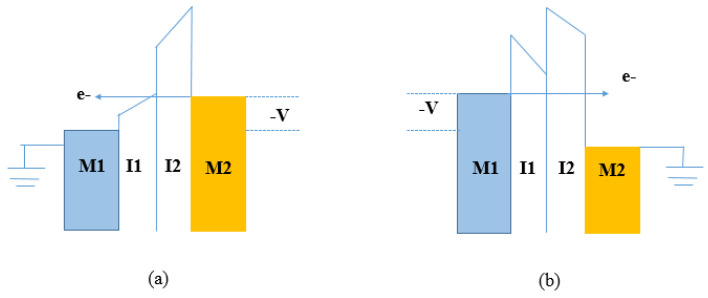
Step tunneling: (**a**) forward bias, (**b**) reverse bias.

In the reverse bias condition in Figure 7b, a negative bias is applied to the higher work function electrode (to the right metal contact M2), whereas the electrode (M1) with the lower work function is grounded. Direct tunneling is active and electrons have to travel through both barriers without any assistance. This results in reduced tunneling probability and, consequently, a lower current density. The QW formed between insulators has to be deep and large enough to form bound quantum states. The TOV, at which bound states form, can be adjusted by varying the thickness of the first insulator; however, this change results in a reduced tunneling current. Figure 8 shows the schematic diagram of the energy band of step tunneling MIIM diode under conditions of forward and reverse bias. In the forward bias condition in Figure 8a, a negative bias is applied to the higher work function electrode (to the right metal contact M2) whereas the electrode (M1) with lower work function is grounded. Electrons tunnel only through the insulator with the widest band gap in one polarity. This implies that the effective tunneling distance decreases with a higher current density. In the reverse bias condition in Figure 8b, a negative bias is applied to the higher work function electrode (to the right metal contact M1), whereas the electrode (M2) with lower work function is grounded. Electrons must pass through both insulator layers, decreasing the current density [7,23,54]. 

### 2.6. THz Diode Technology: State of the Art on Metal Multi-Insulator Metal (MI^n^M) Diode

**Figure 9:**
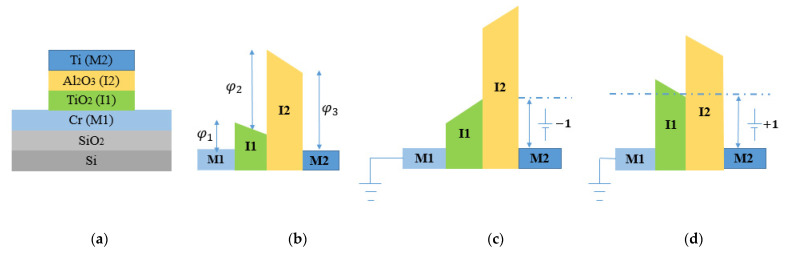

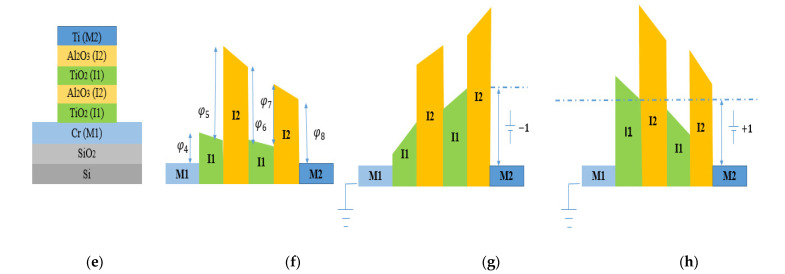
(**a**) Schematic diagrams of the cross-sectional view of the MI^2^M diode. Energy band diagram of the MI^2^M diode: (**b**) no bias applied; (**c**) negative; (**d**) positive bias applied to Ti. (**e**) Schematic diagrams of the cross-sectional view of the MI^4^M diode. Energy band diagrams of the MI^4^M diode; (**f**) no bias applied; (**g**) negative; (**h**) positive bias applied to Ti. Reprinted with permission from ref. [60]. Copyright 2014 Austin Publishing Group.

Various MI^n^M diodes with different combinations of oxide layers (in particular *n* = 2, 3, and 4) and electrodes have been reviewed. FOMs values are obtained, considering selection of materials, thickness of oxides, overlapping area, and fabrication techniques. By considering two or more insulator layers with different electron affinities, the asymmetry and nonlinearity values can influence the behavior of MI^n^M diodes. A study conducted by [59] shows two MI^2^M W/Nb_2_O_5_ (3 nm)/Ta_2_O_5_ (1 nm)/W and W/Nb_2_O_5_ (1 nm)/Ta_2_O_5_ (1 nm)/W able to rectify up to 150 THz. The most relevant results indicate an *S* of 11 V^−1^ at 0.02 V. A study conducted by [60] aims to observe the impact of the number of insulators on performance of the diode. The authors fabricated and characterized a metal-insulator-insulator-metal (MI^2^M), and a metal-insulator-insulator-insulator-insulator- metal (MI^4^M). The selection of metals and insulators is conditioned by parameters such as work functions and difference in the electron affinities. For MI^2^M diode, chromium (Cr) constitutes the first electrode M1, and titanium (Ti) constitutes the second electrode M2. Insulator layers such as TiO_2_ and Al_2_O_3_ are chosen for their difference in the electron affinity. Cr (60 nm) and Ti (100 nm) are deposited on a SiO_2_ substrate. For MI^4^M diode, the used metals are the same as MI^2^M. The total thickness of the insulator layers in the MI^4^M diode is 3 nm, with each single insulator layer thickness of 0.75 nm. Cr/TiO_2_/Al_2_O_3_/Ti for MI^2^M diode and Cr/TiO_2_/Al_2_O_3_/TiO_2_/Al_2_O_3_/Ti for MI^4^M diode are indicated in Figure 9.

**Figure 10:**
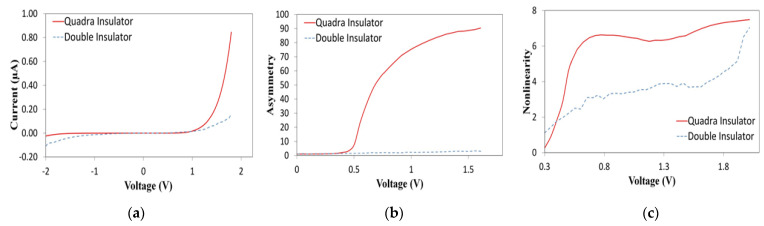
(**a**) Current-Voltage (I-V) curve: (**b**) asymmetry; (**c**) nonlinearity of the MI^2^M and MI^4^M diodes. Reprinted with permission from ref. [60]. Copyright 2014 Austin Publishing Group.

For MI^2^M diode, there are three different interfaces between two metals. Each interface has a different potential barrier value. Figure 9b shows MI^2^M without applying any voltage. The geometry of the energy band diagram is influenced by work functions and electron affinities. In Figure 9c, by applying a bias to one of the metals, the shape of the energy band diagram changes. When a negative bias is applied to the Ti, electron flow occurs from Ti to Cr and is prevented from the opposite side (from Cr to Ti). In Figure 9d, when a positive bias is applied to the Ti, electron flow occurs from Cr to Ti and is prevented from the opposite side (from Ti to Cr). For MI^4^M diode, there are five potential barrier interfaces between the Cr and Ti. Figure 9e shows a schematic diagram of the MI^4^M diode. Figure 9f shows MI^4^M without applying any voltage (zero-bias). Under the bias voltage of −1 V, Figure 9g shows a current higher in the MI^4^M diode than the MI^2^M diode, owing to the difference in the tunneling distances. The same comportment is valuated at +1 V in Figure 9h. MI^4^M diode shows a positive current density higher than the MI^2^M diode. The results observed in Figure 10 indicate, I-V curve, asymmetry, and nonlinearity of the Cr/TiO_2_/Al_2_O_3_/Ti and Cr/TiO_2_/Al_2_O_3_/TiO_2_/Al_2_O_3_/Ti. MI^4^M offers more current than MI^2^M above +1 V bias. At +1.8 V, the current is 0.17 μA for MI^2^M and 0.85 μA for MI^4^M. Figure 10a indicates that MI^4^M diode shows a higher turn-on voltage than that of MI^2^M. In Figure 10b, the *Asym*, is 3 for MI^2^M and 90 for MI^4^M diode at +1.6 V. In Figure 10c, the *NL* for MI^4^M diode is 6 around 0.8 V whereas for MI^2^M, the *NL* is 3.8 at the same voltage. With the same thickness (3 nm), the results indicate a major performance of MI^4^M rather than MI^2^M.

A study conducted by [61] evaluates the performance of Al/Ta_2_O_5_/Al_2_O_3_/Al and Al/Nb_2_O_5_/Al_2_O_3_/Al fabricated on 4 μm × 4 μm glass substrates by considering a thickness between 1 and 6 nm. The results indicate for Al (60 nm)/Nb_2_O_5_ (3–6 nm)/Al_2_O_3_ (1 nm)/Al, a higher current due to reduced band gap and barrier height than Al/Ta_2_O_5_(3–6 nm)/Al_2_O_3_/Al. Al/Ta_2_O_5_/Al_2_O_3_/Al, which shows *Asym* of 18 at 0.3 V, *NL* of 7.5 for a bias < 0.8 V, and *S* of 6 V^−1^ at 0.2 V. Al/Nb_2_O_5_/Al_2_O_3_/Al shows *Asym* of 7.6 for 0 V, *Asym* of 6 at larger voltages, *NL* of 6.8 at voltage < 0.8 V, and *S* of 9 V^−1^ at 0.2 V. In [62], the authors present MI^2^M integrated into a micron-scale antenna for converting MID-IR radiation into electrical power. The diode Co/Co_3_O_4_/TiO_2_/Ti shows an area of 0.071 μm^2^, and it is able to obtain high responsivity at zero-bias voltage and low resistance near zero-bias. These properties make it suitable for EH applications. The diode is constituted by two different metal contacts, Co and Ti, with work functions 5.0 and 4.3 eV, whereas the relative dielectric constants *ε_r_* of 13 and 15–110, respectively, for Co_3_O_4_ and TiO_2_ allow having low diode resistance. The results obtained for an area of 0.071 μm^2^ and from −0.3 to +0.3 V at 222 °C indicate responsivity at 1.2 V^−1^ at zero-bias voltage with a resistance of 14 kΩ. In [63], the authors present MI^2^M with low zero-bias resistance, high responsivity, and insulator layers with a small difference in their electron affinities. 

**Figure 11:**
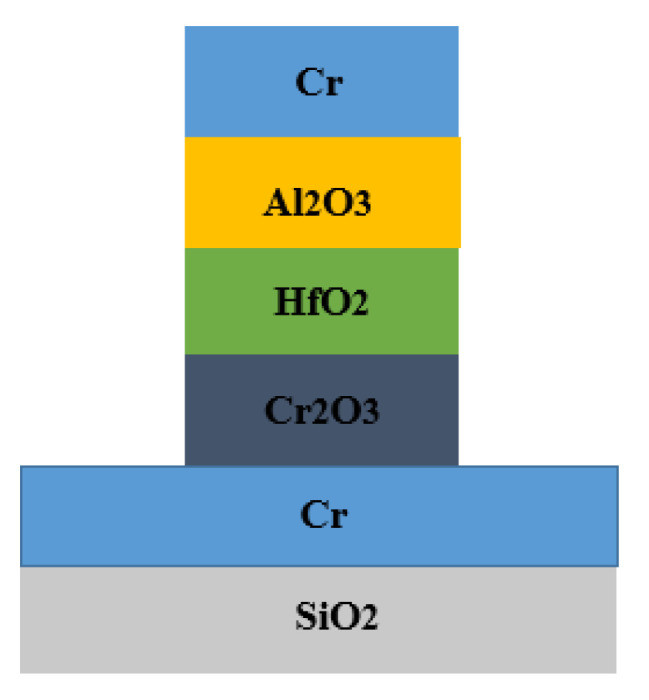
Cross-sectional views of the MI^3^M tunnel device.

The MI^2^M proposed is a Ti/TiO_2_ (1 nm)/ZnO (0.5 nm)/Al, with electron affinities of 3.9 eV and 4.1 eV for TiO_2_ and ZnO and work functions of 4.33 eV and 4.28 eV for the metals Ti and Al, respectively. These metals are excellent candidates for contact pads due to their low barrier heights. With thicknesses fixed at 1 nm for TiO_2_ and 0.5 nm for ZnO and a contact area of 0.01 μm^2^, MI^2^M is integrated on an antenna whose resistance is of 55 Ω. The cutoff frequency obtained by considering a capacitance equal to 16.6 fF/μm^2^ and a time constant of approximately 9.1 fs is of 17.4 THz. The results obtained are zero-bias resistance of 312 Ω with zero-bias responsivity of 1.6 V^−1^ and maximum responsivity of 5.1 V^−1^ at a bias of −200 mV. Diodes governed by electron resonance tunneling mechanism, via multiple quantum wells formation at the oxides interfaces, exhibit extreme asymmetry and high nonlinearity such as ultrahigh speed. In [64], the authors fabricated and characterized two MI^3^M devices consisting of Cr/Cr_2_O_3_/HfO_2_/Al_2_O_3_/Cr and Cr/Cr_2_O_3_/Al_2_O_3_/HfO_2_/Cr. Figure 11 shows the cross-sectional views of the MI^3^M tunnel device. These diodes are made of Cr of 100 nm thick, Al_2_O_3_ insulator layer of 2 nm thick, HfO_2_ insulator layer of 2 nm thick, and Cr_2_O_3_ insulator layer of 2 nm thick. The layers are placed on a 550 nm thick SiO_2_ film on top of a 500 μm thick Si substrate not visible in Figure 11. The oxides are placed sequentially in accordance with their ascending potential barrier values (i.e., 0.64 eV, 0.75 eV, and 2.62 eV, for Cr_2_O_3_, HfO_2_, and Al_2_O_3_, respectively). The results are verified at specific voltage values (−3.0 V, +1.2 V, and +2.7 V, respectively). MI^3^M constrains each well to possess only one allowed state. The *Asym* for Cr/Cr_2_O_3_-HfO_2_-Al_2_O_3_/Cr and Cr/Cr_2_O_3_-Al_2_O_3_-HfO_2_/Cr devices is 5 and 3 around 2.8 V, respectively. 

**Figure 12:**
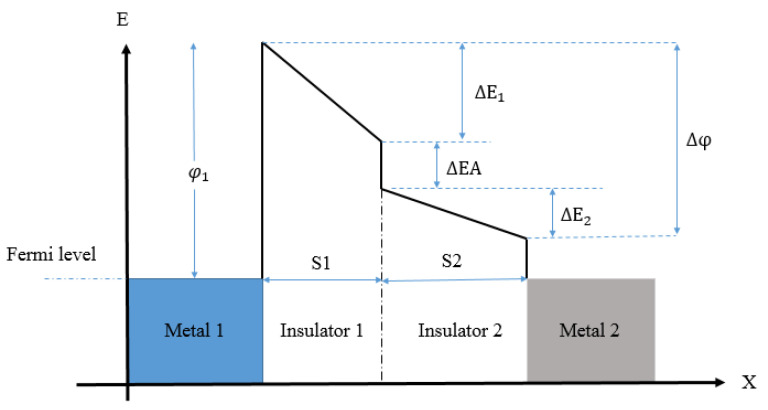
Energy band diagram of the Pt/TiO_2_/TiO_1.4_/Ti. Reprinted with permission from ref. [65]. Copyright 2019 Scientific Reports.

For Cr/Cr_2_O_3_-Al_2_O_3_-HfO_2_/Cr there is a peak 6 around 4.2 V. *NL* for Cr/Cr_2_O_3_-HfO_2_-Al_2_O_3_/Cr is 5 at 1 V and 4 at 1.5 V; at 4.2 V, Cr/Cr_2_O_3_-HfO_2_-Al_2_O_3_/Cr shows a peak equal to 6. In [65], the authors present MI^2^M composed of Pt/TiO_2_/TiO_1.4_/Ti with high current density and high asymmetry simultaneously. Figure 12 shows the energy band diagram of the Pt/TiO_2_/TiO_1.4_/Ti. An oxygen-non-stoichiometric layer, TiO_2−x_, is used as a second layer.

In the schematic, $\varphi_{1}$ is obtained by subtracting the electron affinity of the dielectric from the work function of metal-1, Δ*φ* is the work function difference between metal-1 and metal-2, $\;S_{1}$ and $\;S_{2}$ are the thicknesses of the insulator 1 and insulator 2. ΔEA is the electron-affinity difference between insulator-1 and insulator-2, *ε*_1_ and *ε*_2_ are the respective dielectric constants of the insulators. Δ$E_{1}$ and Δ$E_{2}$ can be expressed by Equation (10) [65].

(10) $$\Delta E_{1}=\frac{S_{1}\varepsilon_{2}}{S_{1}\varepsilon_{2}+S_{2}\varepsilon_{1}}\Delta \varphi ;\;\Delta E_{2}=\frac{S_{2}\varepsilon_{1}}{S_{1}\varepsilon_{2}+S_{2}\varepsilon_{1}}\Delta \varphi$$

**Figure 13:**
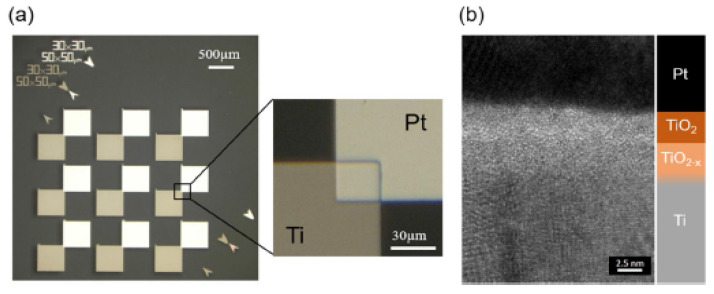
(**a**) MI^2^M fabricated on a silica substrate Si, where the metal pads, Pt and Ti, are overlapped for an area of 900 μm^2^, and (**b**) cross-sectional view of the diode part, obtained by TEM. Reprinted with permission from ref. [65]. Copyright 2019 Scientific Reports.

In forward bias condition, insulator 1 acts as a single tunnel barrier; in reverse bias condition, both insulators function as tunnel barriers, leading to high asymmetry. Pt/TiO_2_/TiO_2−x_/Ti is fabricated on a substrate Si, as shown in Figure 13a. The technique to form the insulator layer is atomic layer deposition (ALD), obtaining high uniformity. 

The metal pads, Pt (50 nm) and Ti (70 nm) are overlapped for an area of 900 μm^2^. The design parameters are *φ*_1_ = 1.7 eV, Δ*φ* = 1.3 eV, dielectric constant *ε*_TiO2_ = 18, and the thicknesses of the oxide layers are *S*_TiO2_ = 3 nm and *S*_TiO2−x_ = 2 nm, respectively. The cross-section in Figure 13b shows the contrast of the upper oxygen-stoichiometric and lower non-stoichiometric oxide layers.

**Figure 14:**
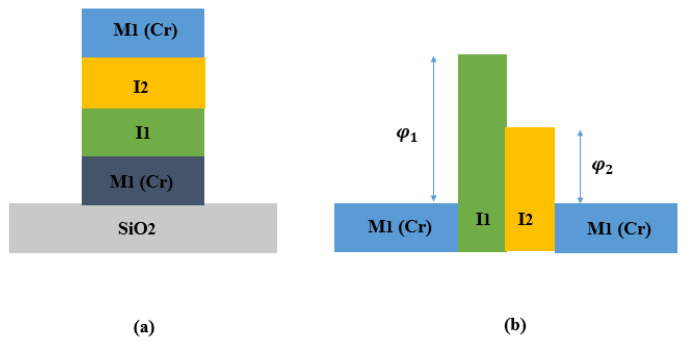
Schematic of (**a**) the layout of the MIIM device and (**b**) the energy band diagram.

The results obtained experimentally for this diode are an average current density at a forward bias of 1 V of 4.2 × 10^6^ A/m^2^ and a maximum asymmetry at 0.45 V of 7.26. By optimizing the film thickness ratio, it is possible to achieve current density *J_ON_* of approximately 10^8^ A/m^2^ with maximum *Asym* of 9. In [66], the authors realized and characterized an asymmetric diode MI^2^M capable of rectifying up to 30 THz. The investigated diode is Cr (100 nm)/Cr_2_O_3_ (3 nm)/Al_2_O_3_ (3 nm)/Ag (100 nm), printed on 500 nm of SiO_2_ substrate deposited on 500 µm Si. The fabrication process for this diode is reported in [66]. The main results obtained in terms of FOMs indicate a density current (J-V) response *J_ON_* of 3 mA/cm^2^ at 0.5 V, and *Asym* >280 at 0.4–0.5 V. In [67], the authors present MI^2^M quantum electronic tunneling devices suitable for high-speed rectifiers. In this context, parameters such as nonlinearity and asymmetry are obtained through cascaded potential barrier architecture and similar metallic electrodes. Considering this, the authors present an operational cascaded potential barrier Cr/Al_2_O_3_-HfO_2_/Cr diode in Figure 14a. Although metal electrodes are of the same material, the cascaded potential barrier profile is asymmetric at zero-bias voltage. In forward bias condition, the electrons pass through a single potential barrier with a high probability of tunneling. In fact, the electrons have to cross the thickness of only one oxide layer and not two oxide layers as in the case of a reverse bias condition. This alteration of tunneling mechanism in forward and reverse bias voltages is attributed to the enhanced nonlinearity peculiar to multi-insulators diode. This diode is constituted by 2 nm Al_2_O_3_ layer and 2 nm HfO_2_ layer, which provides an overall thickness of 4 nm. The potential barrier in Figure 14b is $\varphi_{1}=$ 3.05 eV corresponding to Al_2_O_3_ and $\varphi_{2}=$ 1.75 eV corresponding to HfO_2_. In detail, both electrodes are selected to be of the same metal M1 = M2 = Cr with 100 nm thick Cr layer deposited on a SiO_2_ substrate. 

**Figure 15:**
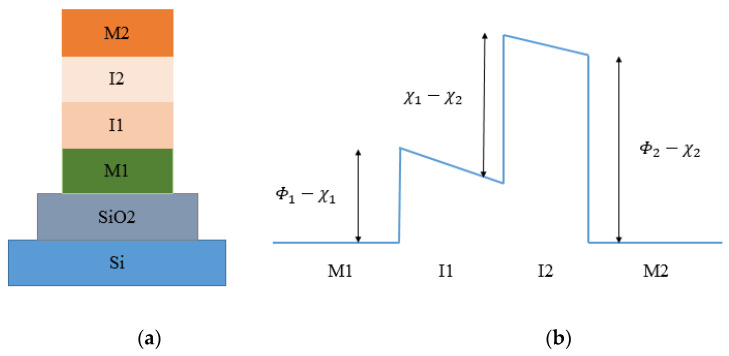
(**a**) Schematic of an M1I1I2M2 diode; (**b**) energy band diagram of M1I1I2M2 diode with different barrier heights. Reprinted with permission from ref. [72]. Copyright 2021 IOP Publishing.

The results indicate in the forward direction (*V* > 0) a current of 64 μA at *V* = 3V and in the reverse direction at *V* = −3 V only 7 μA. Although the diode is made of the same metals, the diode shows high asymmetry as the oxide layer is modified to form a cascaded potential barrier layer; therefore, the barrier height symmetry is broken and asymmetric I-V behavior is observed. The results indicate an *Asym* of 1.5 around zero-bias voltage and an *Asym* of 10 around 3 V. The *NL* is of 4 around 1 V and of 10 around 2.3 V. The addition of another insulator layer in the MIM configuration provides an increase in asymmetry and nonlinearity. In forward bias condition, resonant tunneling leads to great conductivity while maintaining nonlinearity. In reverse bias condition, step tunneling results in high asymmetry with a sharp turn-on. In [68], the authors realized and characterized an asymmetric diode MI^2^M capable of rectifying up to 30 THz. The diode is ZCAN (ZrCuAlNi 150 nm)/HfO_2_ (5 nm)/Al_2_O_3_ (3 nm)/Al (150 nm), printed on 100 nm of SiO_2_ substrate deposited on Si substrate. The fabrication process for this diode is reported in [68]. The main results obtained in term of FOMs indicate an *Asym* > 10 at 0.8 V, and a *NL* > 5 at 0.8 V. In [69,70], the authors realized and characterized one asymmetric diode MI^2^M capable of rectifying up to 30 THz. The diode is Pt (150 nm)/HfO_2_ (1.5 nm)/TiO_2_ (1.5 nm)/Ti (150 nm), with 20 μm × 20 μm junction area, printed on 500 nm of SiO_2_/Si substrate. The fabrication process for this diode is reported in [69,70]. The main results obtained in term of FOMs indicate an *Asym* of 10 at 0.8 V, *NL* > 5.5 at 0.8 V, peak responsivity *S* (V^−1^) of 2 × 10^4^, and zero-bias differential resistance of 0.1 MΩ. This diode is particularly suitable for EH applications due to a very small *V_bias_*. In [69], authors realized and characterized one asymmetric diode MI^2^M capable of rectifying up to 30 THz. The diode is Pt (150 nm)/Al_2_O_3_ (1.5 nm)/TiO_2_ (1.5 nm)/Ti (150 nm), with 20 μm × 20 μm junction area, printed on 500 nm of SiO_2_ substrate deposited on Si substrate. The main results obtained in term of FOMs indicate an *Asym* of 17 at 5 V, *NL* > 5.5 at 5 V, peak responsivity *S* (V^−1^) of 2 × 10^4^, and zero-bias differential resistance of 0.1 MΩ. Due to a very high *V_bias_*, this diode is not suitable for EH applications. In [71], the authors present a double insulator Ni–NiO_x_–ZnO–Cr tunnel diode. The surface roughness of the complete NiOx and ZnO stack is 3.2 nm over an area of 20 μm × 20 μm. The turn-on voltage for this diode is nearly 250 mV. The band gap value of the NiO_x_ is found to be 3.04 eV, while the band gap of ZnO at room temperature is 3.37 eV. The performance of two thin-film insulators is influenced by barrier heights, bias polarity, surface roughness of metal electrodes, uniformity of the dielectric, and junction area. Tunneling probability of the electrons increases with the decreasing insulator thickness but at the cost of asymmetry and nonlinearity. The work functions of Ni and Cr are 5.15 eV and 4.5 eV, respectively, whereas the electron affinity of NiO_x_ is of 0.6 eV. The barrier height difference is of 0.65 eV. The effective barrier thickness of 3 nm across the 20 μm × 20 μm contact area is lower than the targeted thickness of 7 nm. The results obtained are an *Asym* of 16 at 0.5 V and a current of 1.8 × 10^−7^ A at 0.5 V. Finally, in [72], the authors carried out a simulation study of the multi-junction insulator tunnel diode for solar energy harvesting applications. Specifically, in this article, the idea advanced by the authors [72] is to study various possible combinations of oxide materials and optimized thickness with fixed electrodes to obtain a high operating cutoff frequency (28.3 THz or 10.6 μm). The authors’ efforts are concentrated on considering only two insulators. Platinum (Pt; work function = 5.65 eV) as bottom metal and titanium (Ti; work function = 4.33 eV) as top metal for M1I1I2M2 diode were selected to obtain a high work function difference. In Figure 15a, the schematic of M1I1I2M2 is shown, where M1 and M2 represent the metals at the bottom and top and I_1_ and I_2_ represent the different oxide layers of the diode. Figure 15b represents the energy band diagram of an M1I1I2M2 diode with different barrier heights. 

These two insulators in Figure 15b have different electron affinities, which results in a discontinuity at their interfaces. Table 4 shows different combinations of oxide layers and optimized thickness. 

**Figure 16:**
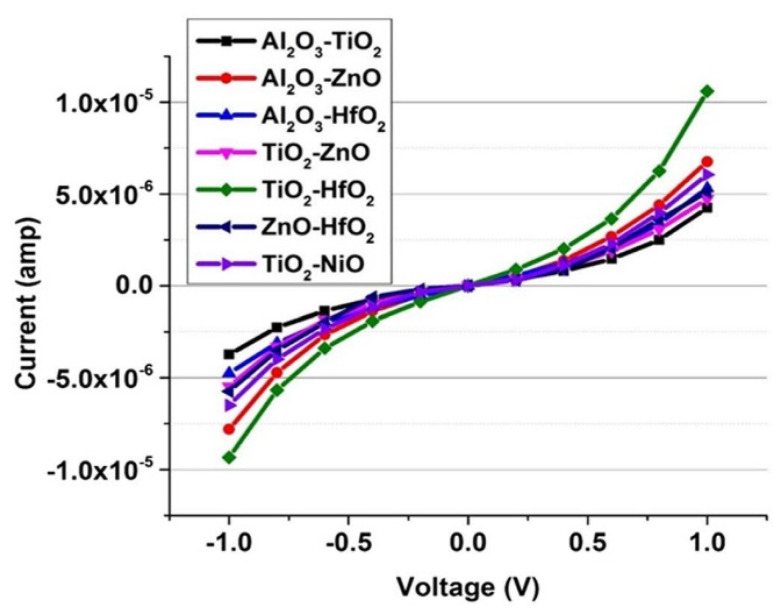
The I-V response of diode for different oxide combinations. Reprinted with permission from ref. [72]. Copyright 2021 IOP Publishing.

In the simulation model, the authors consider an overlap area of 100 nm^2^, a standard antenna impedance of 50 Ω, and a voltage sweep range set from −1 to 1 V. Figure 16 shows I-V response of diode for different oxide combinations.

**Figure 17:**
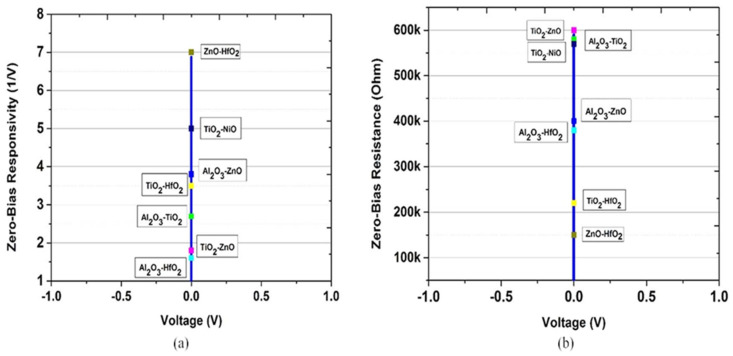
(**a**) The zero-bias responsivity of diode for different oxide combinations; (**b**) the zero-bias resistance of diode for different oxide combinations. Reprinted with permission from ref. [72]. Copyright 2021 IOP Publishing.

The zero-bias resistance and zero-bias responsivity are indicated in Figure 17a,b. In Table 5, the results for the oxide combination are reported. The difference in the electron affinities between the two insulator layers leads to an extra barrier at the interfaces of the oxide, which increases the asymmetry and the nonlinearity. 

The results indicate that insulators with a significant difference in electron affinity enhance the responsivity of the M1I1I2M2 diode. The recent studies on MI^n^M discussed in this subsection are summarized in Table 6.

## 3. THz Diode Technology: General Information on Geometric Diode (GD)

In light of the above discussion, a planar structure designed to operate at the THz frequency must meet two requirements, an extremely low RC time constant and a high coupling efficiency (the real part of the impedance of the diode has to be relatively close to that of the nanoantenna). These requirements cannot be obtained simultaneously; in fact, the reduction of the overlapping area of diode involves a low *C* whereas R increases proportionately. 

**Figure 18:**
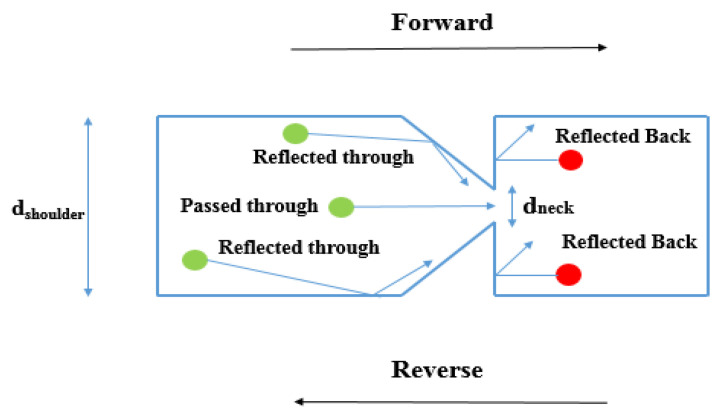
A schematic of the graphene geometric diode (GD).

A completely different approach is a device referred to as a geometric diode. It shows rectification properties thanks to its asymmetric structure that induces different current levels for forward and reverse motion [73]. The asymmetric structure observable in Figure 18 forces one part of the charge carriers to flow in one direction only from left to right through *d_neck_*, whereas a second part of the charge carriers will be blocked and reflected away from *d_neck_* constriction [7,19,20,23,24]. 

During the design phase, the diode must meet some requirements. Long mean-free path length (MFPL) is the average distance that an electron travels before substantially changing its direction due to one or more successive collisions with other electrons, atoms, or defects. If MFPL is short, a faster response time is verified; however, as MFPL decreases, the collision time increases. When the condition *d_neck_* < MFPL is verified, the electrons can move ballistically and the probability that the electrons pass in reverse direction will decrease significantly, obtaining a high rectification performance [74]. As *d_neck_* increases, electrons can move more easily through the constriction with an increase in the magnitude of the current. Another design parameter is *d_shoulder_.* If it increases, an increase in current through *d_neck_* and an increase in the asymmetry are observed simultaneously. However, above a certain shoulder width, both the current and the asymmetry change. Therefore, a larger *d_shoulder_* will increase the forward current and a smaller *d_neck_* will reduce the reverse current [14,55,56]. Due to asymmetry in the structure, no external voltage bias is applied (zero-bias turn-on voltage), making this device adequate for EH applications. This planar structure presents both low resistance and ultra-low capacitance, overcoming the capacitance/resistance tradeoff. The metals possess a high current density *J_ON_* up to 10^7^ A/cm^2^; however, the MFPL is only a few nanometers, approximately from 10 to 60 nm at room temperature. A good choice to fabricate this diode type is represented by graphene, which, when placed on a silicon oxide substrate SiO_2_ above a substrate Si layer, shows current density *J_ON_* up to 10^8^ A/cm^2^ and MFPL up to 1 μm. In addition, the graphene allows the electrons to move freely in the plane without encountering scattering points on the contrary in metals, where electrons move through a 3D plane encountering scattering points, impurities, and thermal vibrations. However, 2D material requires ultrafine lithography to form the device and this makes the manufacturing process challenging [75,76,77,78,79,80,81,82].

### THz Diode Technology: State of the Art on Geometric Diode (GD)

Although there have been continuous improvements, the current state indicates that there is no extensive and detailed literature on this type of diode. Only a few authors have provided a significant improvement over the state of the art. This paragraph presents a review of the most recent progress and the latest results and issues for this asymmetric structure. In [83], the authors present an infrared optical response of geometric diode rectenna solar cells. The geometric diode is an inverse arrowhead-shaped graphene device coupled to bowtie antenna (5.1 μm × 2.3 μm). GD was fabricated with the e-beam lithography (EBL) technique. Cr/Au metal contact pads formed a four-point probe configuration. Design parameters indicate a neck width of 70 nm, a shoulder width of 400 nm, and MFPL estimated around ~50 nm. With a capacitance of the order of 1 aF and an RC time constant of ~1.6 fs, this device has achieved an operating frequency of 28.3 THz. A current *I_DS_* (μA) was measured as a function of drain-source bias *V_DS_* (V). For a gate voltage *V_G_* = 20 V and a *V_DS_* = 1.5 V, 150 μA is obtained. Zero bias resistance measured as a function of drain-source bias *V_DS_* (V) indicates 13 KΩ for *V_DS_* = 0 V. Finally, responsivity measured as a function of the input AC power indicates 0.18 V^−1^ for *V_DS_* = 0 V and 0.2 V^−1^ for *V_DS_* = 1.5 V. In [84], the authors present an optical rectenna solar cell using graphene geometric diodes. The authors fabricated this device using graphene obtained by exfoliation that adheres to a 90 nm thick oxidized silicon wafer. The only design parameter is a *d_neck_* = 150 nm. This diode has the advantage of having very low capacitance of the order of 0.06 fF due to its planar structure with a low RC time constant of ~7 fs, allowing an operating frequency in the optical range. The results indicate for *I_DS_* (μA), measured as a function of *V_DS_* (V), a peak around 0.01 μA for *V_DS_* = 0 V. For zero-bias resistance measured as a function of *V_DS_* (V), there is a peak around 19 KΩ for *V_DS_* = 0 V. The responsivity *S* measured as a function of *V_DS_* (V) indicates a peak around 0.3 V^−1^ for *V_DS_* = 0 V and a peak around 0.8 V^−1^ for *V_DS_* = 0.4 V. By keeping all other parameters fixed, *I_DS_* (mA) and asymmetry are obtained as a function of *d_neck_* (50, 200, and 600 nm). The best values are obtained for *d_neck_* = 600 nm and *d_neck_* = 50 nm. In fact, *I_DS_* (mA) of 1.5 mA is obtained in correspondence of *V_D_* = 0.1 V and *d_neck_* = 600 nm, whereas an asymmetry of 3.2 is obtained in correspondence of *V_D_* = 0.05 V and *d_neck_* = 50 nm. In [85], the authors present graphene geometric diodes for terahertz rectennas. This device, although designed to resonate at 28 THz, provides low resistance and a low RC time constant of 10^−15^s, required for operation at optical frequencies. For *V_DS_* = 0.1 V and *d_neck_* = 600 nm, *I_DS_* (mA) assumes the maximum value of 1.5 mA. For *d_neck_* = 75 nm, *d_shoulder_* = 400 nm, and an MFPL = 45 nm, the diode obtains a responsivity *S* of 0.12 V^−1^ at zero-bias voltage, and a responsivity over 0.2 V^−1^ around *V_DS_* (V) =1.4 V. *I_DS_* (μA) of 0.01 at zero-bias voltage, and an *I_DS_* (μA) over 150 at *V_DS_* (V) = 1.4 V are obtained. 

Other results indicate a rectifying antenna coupling efficiency of 12% with diode resistance of 3 KΩ, an antenna impedance of 100 Ω, and an antenna radiation efficiency of 37%. In addition, quantum efficiency of 0.01% and zero-bias responsivity of 0.0285 V^−1^ are obtained. All diodes discussed above are made by considering a monolayer graphene obtained by the exfoliated graphene process. This technique, however, presents two inconveniences.

**Figure 19:**
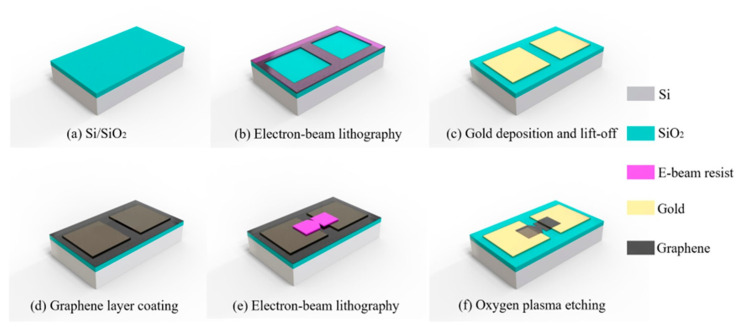
The fabrication process of the graphene geometric diode. Reprinted with permission from ref. [86]. Copyright 2021 Nanomaterials.

First, graphene can be realized in a very small area through the exfoliation process; this makes it not suitable for mass production. Second, the exfoliated graphene is not uniform; therefore, some areas of its surface, may be multi-layer rather than monolayer. In [86], the authors present a CVD-grown monolayer graphene-based geometric diode for THz rectennas. This process allows realizing rectennas at a mass scale. The fabrication process of the graphene starts in the cleanroom through high-resolution EBL processes, as shown in Figure 19. A 300 nm layer of SiO_2_ is deposited on a highly doped Si wafer through a plasma-enhanced (PE)—chemical vapor deposition (CVD) (Figure 19a). On SiO_2_, a layer of e-beam resist ZEP520A is deposited. ZED-N50 was used to form two square-shaped trenches (Figure 19b). A Ti (10 nm)/Au (40 nm) layer was deposited on the sample through sputtering. The two contact pads were realized through the lift-off process (Figure 19c). The monolayer graphene was transferred on the sample with Ti/Au electrodes (Figure 19d). In the gap between the metal pads (Figure 19e), an e-beam resist PMMA 950 K A4 was spin-coated and patterned by EBL. The last step indicates an oxygen plasma etching utilized to realize the graphene geometric diode (Figure 19f). 

**Figure 20:**
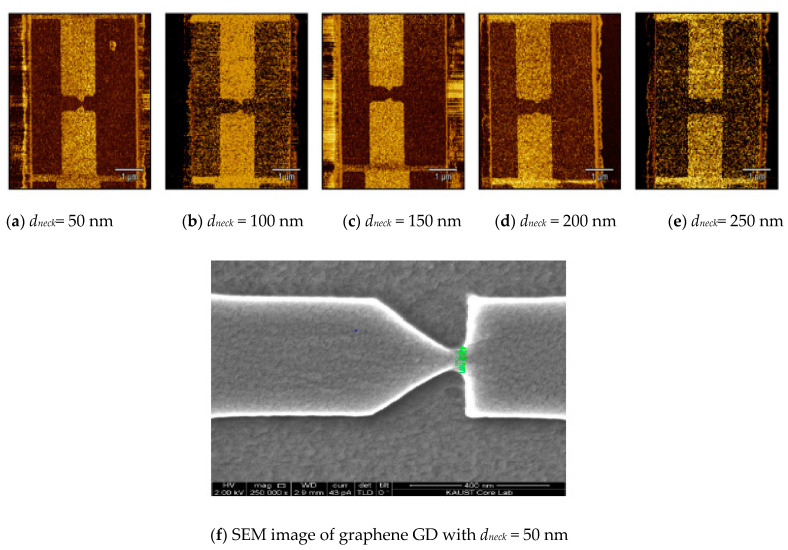
AFM images of the graphene geometric diode (the black area is graphene) with different neck widths: (**a**) 50 nm neck width, (**b**) 100 nm neck width, (**c**) 150 nm neck width, (**d**) 200 nm neck width, (**e**) 250 nm neck width, (**f**) SEM image of the 50 nm neck width geometric diode. Reprinted with permission from ref. [86]. Copyright 2021 Nanomaterials.

To verify the fabrication process of graphene GD, atomic force microscopy (AFM) was used with different *d_neck_* as shown in Figure 20. The increase of *d_neck_* is observed in the AFM images in Figure 20a–e. Figure 20f shows a graphene GD with *d_neck_* = 50 nm. 

**Figure 21:**
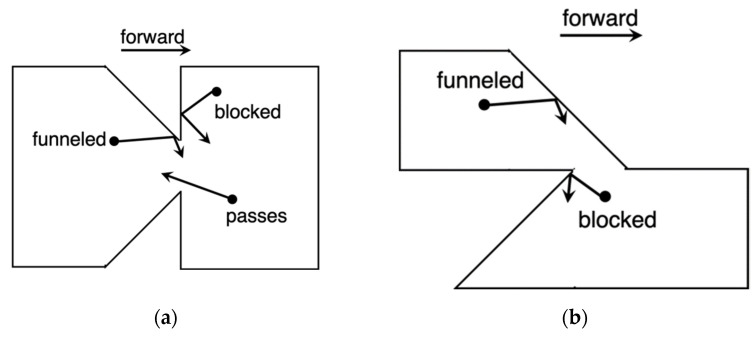
(**a**) Geometric effect in the inverse-arrowhead diode; and (**b**) geometric effect in the Z-diode. Reprinted with permission from ref. [87]. Copyright 2021 Nanomaterials.

*I_DS_* (mA) is measured as a function of *V_DS_* (V) by varying *d_neck_* from 50 nm to 250 nm and *V_DS_* from −2 V to 2 V. For *d_neck_* = 50 nm and *V_DS_* (V) = 2, *I_DS_* (mA) reaches a maximum of 0.7. For *d_neck_* = 100 nm and *V_DS_* (V) = 2, *I_DS_* (mA) reaches a maximum of 1.3. For *d_neck_* = 150 nm and *V_DS_* (V) = 2, *I_DS_* (mA) reaches a maximum of 1.4. For *d_neck_* = 200 nm and *V_DS_* (V) = 2, *I_DS_* (mA) reaches a maximum of 1.5 and finally, for *d_neck_* = 250 nm and *V_DS_* (V) = 2, *I_DS_* (mA) reaches a maximum of 1.8. For *V_DS_* fixed, *I_DS_* (mA) increases as *d_neck_* increases. This is due to the greater flow of charges that carriers can pass through *d_neck_* per second. It is observed that parameters such as nonlinearity and asymmetry that identify the performance of the diode begin to decrease as *d_neck_* increases. For *V_DS_* = 2 V, *d_neck_* = 50 nm, and *d_neck_* = 100 nm, the *Asym* obtained is 1.40 and 1.25, respectively. For the other *d_neck_* values, the *Asym* is equal to 1; therefore, no rectification is observed. Zero-bias resistance and zero-bias responsivity tend to decrease as *d_neck_* increases. The results indicate that zero-bias resistance (Ω) reaches a maximum of 5 × 10^3^ or *d_neck_* = 50 nm, and a minimum of 2 × 10^3^ for *d_neck_* = 250 nm. Zero-bias responsivity (V^−1^) reaches a maximum of 0.06 for *d_neck_* = 50 nm, and a minimum of 0.02 for *d_neck_* = 250 nm. Finally, when a small *V_DS_* is applied to GD, the resistance is reduced, and the responsivity is increased. For a fixed *d_neck_* = 50 nm and *V_DS_* (V) = 0, the resistance (Ω) is 5 × 10^3^; instead, for *V_DS_* (V) = 0.5 and d_neck_ = 50 nm, the resistance is 4 × 10^3^. The responsivity *S* (V^−1^) assumes 0.1 for *V_DS_* (V) = 0 and 0.3 for *V_DS_* (V) = 0.5. In [87], the authors present a new geometric diode design, referred to as Z-diode, exhibiting improved rectification over 0.1 V compared to the inverse-arrowhead design discussed above. For this type of diode that is able to rectify up to 28 THz, a finite mean-free-path length (MFPL) does exist that maximizes the current asymmetry at a particular voltage. This suggests that the diode can be tuned to maximize the asymmetry. In both structures, inverse-arrowhead (Figure 21a) and Z-diode (Figure 21b), under forward bias condition, electrons drift from left to right, and the geometry allows electrons to pass through the constriction, or neck.

**Figure 22:**
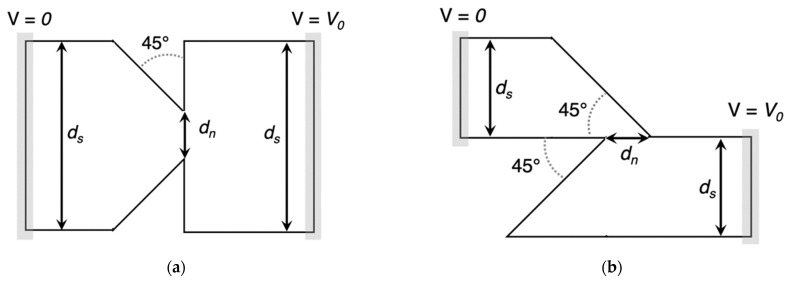
(**a**) Relevant dimensions of an inverse-arrowhead diode; and (**b**) a Z-diode. Reprinted with permission from ref. [87]. Copyright 2021 Nanomaterials.

However, in the reverse bias condition, these two structures present a substantial difference. In fact, for inverse-arrowhead geometry, electrons drift right to left, more easily through the neck, as the opening is perpendicular to the general drift direction. In the Z-diode, the reverse leakage is reduced as the neck opening is oriented perpendicular to the general drift direction. The strength of the geometric rectification effect depends on the device dimensions relative to *λ*_MFPL_. To obtain the I(V) characteristics, both structures used *λ*_MFPL_ = 500 nm, neck width *d_n_* = 100 nm, and shoulder width *d_s_* = 1 µm, indicated in Figure 22a,b. The shoulders are electrodes from which charges are injected. Voltage *V*_0_ from −0.5 to 0.5 V is applied to the electrodes to drive the current through the device. 

**Figure 23:**
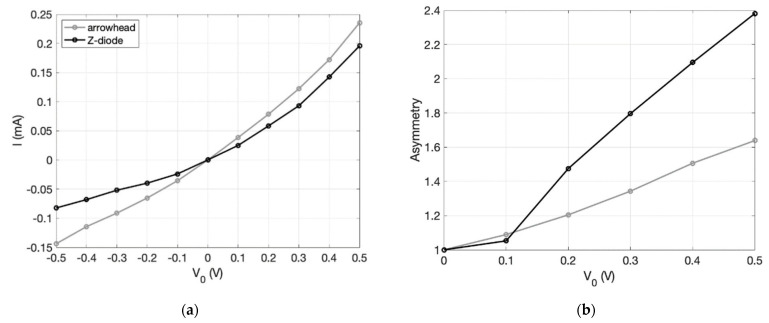
Comparison of inverse-arrowhead diode and Z-diode. (**a**) The current–voltage characteristics of both geometries; and (**b**) the resulting current asymmetries. Reprinted with permission from ref. [87]. Copyright 2021 Nanomaterials.

The material used for simulations is graphene, which can be tuned by applying a gate bias set at *V_g_* = 10 V for the simulations. The graphene subsequently is placed on silicon wafers. In Figure 23a,b, the Z-diode exhibits a greater nonlinear I(V) curve and higher current asymmetry above 0.1 V compared to inverse-arrowhead diode. This is due to the stronger suppression of the reverse current owing to the geometry. Despite these results, Z-diode shows higher resistance at low biases, compared to the inverse-arrowhead diode. This factor must be considered when the diode is integrated to the antenna. 

**Figure 24:**
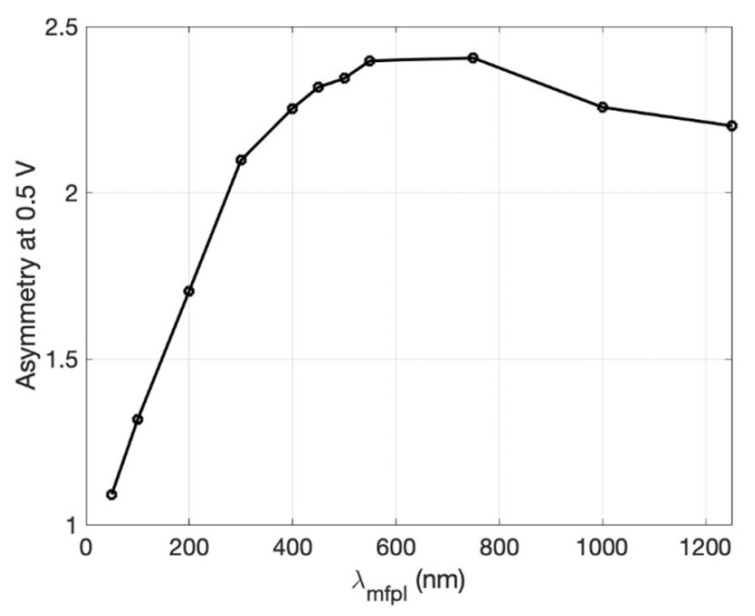
Asymmetry dependence on carrier mean-free-path length for a graphene Z-diode. Reprinted with permission from ref. [87]. Copyright 2021 Nanomaterials.

In Figure 24, the asymmetry as a function of *λ*_MFPL_ is shown. As *λ*_MFPL_ approaches zero, the asymmetry approaches 1.

With *d_neck_* = 100 nm, the asymmetry in Figure 24 shows a peak around *λ*_peak_ ≈ 600 nm, then asymmetry drops for *λ*_MFPL_ *> λ*_peak_. The latest studies on graphene GD reviewed in this paragraph are summarized in Table 7.

## 4. Perspectives in THz Rectifier Technology for Energy Harvesting Applications

The widespread use of rectennas in RF and microwave range has suggested the inspiration for extending the concept of rectenna to THz, from infrared (IR) to solar optical frequencies [4]. The rectenna tuned at IR frequency can also be employed at night and has no particular efficiency limits [88]. Efficiencies above 85% at microwave frequencies have been observed in the laboratory; however, as the frequency increases, the device cannot simply be scaled down, due to the significant changes in material properties; consequently, the efficiency will be very low [4]. Schottky diodes are currently the building block in THz technologies up to 5 THz [33,81]; however, as the frequency increases, this diode becomes slow [89]. Technological maturity has already been reached for this type of diode, so now no major changes can be expected with the current technological process to reduce the problems of series resistance and parasitic capacitance [90,91,92,93]. Therefore, if the current trend continues, the diodes examined in this review, based on the principles of quantum tunneling effect [4], ballistic and quasi-ballistic transport [87], will have the possibility of surpassing the Schottky diode. The future of these diodes will be determined by the ability to improve their efficiency. The future of THz rectifier technology for EH applications promises to be intriguing. The main impact is that it will become possible to harvest and convert low-level energy that cannot be harvestable today. The possible perspectives advanced by the authors are highlighted as follows.

### 4.1. MIM and MI^n^M Diodes

Although nanoantenna technology presents significant challenges in terms of DC power requirements, conversion efficiency, parasitic interactions between antennas, development of miniaturization of new materials, and alternatives to noble metals, potential topics are represented by the fabrication and characterization of rectifier diodes operating at THz frequencies. 

The future of this type of diode promises to be intriguing even though they are not currently commercially available at a large scale. This technology, due to its ability to operate at zero-bias, will have a major impact on EH applications. Although the literature indicates the value of 343 THz [48] as the best cut-off frequency, intensive work is still necessary to improve the performance of these nonlinear devices. Single-insulator (MIM) and multiple insulator (MI^n^M) could represent in the near future a possible candidate for rectenna at THz frequencies. However, several issues discussed above are associated with the possible fabrication of these diodes. Currently, the trend focuses on several areas of investigation. The first area of investigation is the manufacturing process, which in the next few years could contribute to overcoming the impedance mismatch antenna-diode. Different combinations of materials could be the second area of investigation to obtain the desired I-V characteristic and high FOMs. The requirement for highly precise manufacturing processes, metal contact deposition, ability to grow or deposit ultrathin oxide layers, and patterning nanoscale devices represent the third area of investigation. Among the various methods of oxides deposited directly on the metal, ALD offers the best quality oxides with very precise thickness control. However, it is a very expensive and time-consuming process. Therefore, other less expensive methods used for this purpose could be native, thermal, anodic, and plasma oxidation. The use of scanning electron microscopy (SEM) and transmission electron microscopy (TEM) allows us to observe the morphology of the deposited oxides [28]. Finally, the last area of investigation is how to improve the coupling efficiency. In this direction, high FOMs with low resistance can be simultaneously obtained by adding a second insulator layer (MI^2^M) or more insulator layers (MI^n^M) [4,59,94,95,96]. 

### 4.2. Geometric Diode (GD)

Based on a planar structure, this type of diode shows both low resistance and ultralow capacitance. This technology is still in its infancy and several limitations are waiting to be overcome. For example, for rectification to occur, charge carriers must travel ballistically (MFPL greater than device size) or quasi-ballistically (MFPL close to device size) [86,87] through the material, which requires that the sizes of the device should be approximately MFPL or lower. This places stringent manufacturing requirements on conventional metal-based devices, where typical mean-free-path lengths are less than 100 nm. This difficulty can be overcome by using 2D materials, such as graphene for its mean-free-path lengths on a micron scale. A new geometric diode designed at 28.3 THz, the graphene Z-diode, shows improved rectification of more than 0.1 V compared to the previous inverse-arrowhead design. The possible perspectives advanced by these authors are divided into two areas. The first area of investigation is focused on how to achieve this fabrication process at a low cost. The second area of investigation is the selection of new materials because as the frequency increases, metals become very resistive. The results obtained using this type of diode around 30 THz are more encouraging [86,87,97,98,99,100,101,102,103].

## 5. Conclusions

In this review, several aspects of THz diode technology have been discussed. Rectennas are considered a complementary solution to solar cells due to their various discussed advantages. However, although significant improvements in the design and fabrication process have been seen in recent years, the diode rectifier remains the most critical part of the system, with a maximum operating frequency of 343 THz and efficiency lower than 1%. Furthermore, the compatibility with the desired figures of merit still remains an open question. Currently, the use of a rectenna has only been successfully demonstrated for harvesting microwave and radio frequency. In light of the results discussed above, it is not possible to obtain a rectifying diode able to rectify frequencies at hundreds of THz with the current technology. However, we find that the results obtained for the infrared-frequency rectifying diode around 30 THz are more encouraging. Therefore, a new generation of diodes with small resistance values must be developed to transfer more power to the load. This will require significant creativity and efforts in the design, materials, and manufacturing processes.

## Figures and Tables

**Table 1:** Summary of benefits and common issues for planar diodes at THz frequencies.

Parameter	Description	Benefits	Common Issues
*η*	Overall efficiency	Up to 100% under monochromatic illumination; Up to 44% under broadband solar illumination	The real total efficiency is around 0.001% due to: choice of materials and antenna design; antenna/diode mismatch; very low responsivity
*J*	Tunneling-current density	Able to convert AC to DC	An insulator layer with a thickness less than 3 nm remains challenging
$f_{C}$	Cut-off frequency	At present the maximum cut-off frequency is 343 THz	$\mathrm{Very}\;\mathrm{low}\;\tau_{D}$ involves thickness of the insulator layer beyond 5 nm which reduces the tunneling current and increases the diode resistance
Asym	Asymmetry	Asym>1 indicates rectification in the diode	An insulator layer with a thickness less than 3 nm remains challenging
*NL*	Nonlinearity	NL>3 indicates the measure of the deviation from a linear resistor	As the insulator layer increases, the value of the tunneling current decreases and diode resistance increases
*S*	Responsivity	If S>3 the diode rectifies the signal efficiently	An increase in high barrier involves large resistance
$R_{D}$	Diode resistance	$\mathrm{If}\;R_{A\;}$$=R_{D}$ it involves: maximum transfer of the energy captured by the antenna toward the load; high tunneling-current density	If the resistance value is very low, the diode shows very low values for responsivity and non-linearity
TOV	It represents the positive voltage in order to “turn on“ the diode	TOV has a low value for thin insulator layer	Very low nonlinearity
MIM diode	Ultrathin insulator layer in between two dissimilar metal contacts	The current state of the art indicates rectification signals up to 343 THz	Higher FOMs and low resistance cannot be obtained with a single insulator layer
MI^2^M or MI^n^M diodes	Multiple insulator layers sandwiched in between two similar or dissimilar metal contacts	Higher efficiency compared to MIM; higher FOMs and low resistance compared to MIM; similar metals can also be used as contacts	Higher bias to move the electrons out of the quantum well (QW); by inserting two or more insulator layers, the tunneling resistance becomes high, by reducing the current.

**Table 2:** MIG configurations and their contributions.

Material	*J_ON_*	*Asym*	*NL*	*S* (V^−1^)
Al/AlO_x_/Gr [23]	1.0 A/cm^2^ @ ± 1 V	2500 @ ± 1 V	3.8	-
Gr/TiO_2_/Ti [42]	7.5 A/cm^2^ @ ± 1 V	520	15	26
Ti/TiO_2_/Bilayer Graphene [43]	0.1 A/cm^2^ @ ± 1 V	9000	8	10
Gr-h-BN-Gr [44]	0.02 A/cm^2^ @ ± 1 V	1000	40	12

**Table 3:** Summary of recent achievements in the field of MIM diode for THz rectennas.

Material	Cut-Off Frequency	Thickness	*J_ON_*	*Asym*	*NL*	*S* (V^−1^)	Zero-Bias *S* (V^−1^)
Cu (100 nm)-CuO-Au (100 nm) (0.0045 μm^2^) [8]	28.3 THz	CuO (0.7 nm) Au/Cu (100 nm)	-	-	-	6	4
Ti-TiO_2_-Al (21,287 µm^2^) [45]	Up to 150 THz	TiO_2_ (9 nm)	10^−1^ A/cm^2^	-	6.5	18	-
Ti-TiO_2_-Pt (21,287 µm^2^) [45]	Up to 150 THz	TiO_2_ (9 nm)	10^−0^ A/cm^2^	-	15	15	-
Nb/Nb_2_O_5_/Pt [46]	Up to 150 THz	Nb_2_O_5_ (15 nm)	-	1500	4	20	-
Nb/Nb_2_O_5_/Cu [46]	Up to 150 THz	Nb_2_O_5_ (15 nm)	-	1500	8	20	-
Nb/Nb_2_O_5_/Ag [46]	Up to 150 THz	Nb_2_O_5_ (15 nm)	-	1500	8	20	-
Nb/Nb_2_O_5_/Au [46]	Up to 150 THz	Nb_2_O_5_ (15 nm)	-	1500	8	20	-
Au/Al_2_O_3_/Pt [47]	Up to 28.3 THz	Al_2_O_3_ (1.4 nm) Au/Pt (100 nm)	-	-	6	-	10
Ni-NiO-Ag (3.1 × 10^−4^ µm^2^) [48]	Up to 343 THz	NiO (6 nm)	-	5	3	8.5	5.8
Pt-SiCl_3_-(CH_2_)_17_-CH_3_-Ti (100 μm^2^) [49]	Up to 150 THz	SiCl_3_-(CH_2_)_17_-CH_3_ (2.23 nm)	-	117.8	6.8	20.8	8.0
Nb/TiO_2_/Pt [29]	Up to 30 THz	TiO_2_ (13 nm)	-	80	3.5	-	-
Nb/Nb_2_O_5_/Ni [29]	Up to 150 THz	Nb_2_O_5_ (15 nm) Nb/ Ni (90–100 nm)	1 × 10^−10^ A/cm^2^	396.5	7.1	8.5	-
Nb/Nb_2_O_5_ (15 nm)/Au [27]	Up to 150 THz	Nb_2_O_5_ (15 nm) Nb/Au (90–100 nm)	-	1430.8	8.0	7.0	-
SrTiO_3_ (STO)/Al_2_O_3_/SrTiO_3_ (STO) [50]	Up to RF	-	5 × 10^−9^ A/cm^2^	-	-	-	-
Cu-CuO-Cu (2 × 2 μm^2^) [51]	Up to 150 THz	CuO (2 nm) Cu (100 nm)	-	-	-	4.497	-
Pt/Al_2_O_3_/Al [52]	Up to 150 THz	Al_2_O_3_ (6 nm) Pt/Al (100 nm)	-	110 for AP-CVD 30 for PEALD	6 for AP-CVD 30 for PEALD	9 for AP-CVD 22 for PEALD	-
Al-Al_2_O_3_-Au [53]	Up to 60 THz	Al/Au (65 nm)	4.0 μA/cm^2^	-	-	14.46	-
Al-Al_2_O_3_-Cr [19]	Up to 28.3 THz	Al_2_O_3_ (3 nm) Al /Cr (100 nm)	2 × 10^−4^ A/cm^2^	-	3.1	-	-

**Table 4:** Different combinations of oxide layers and optimized thickness to match the peak intensity frequency range. Reprinted with permission from ref. [72]. Copyright 2021 IOP Publishing.

Oxide	Dielectric Constant	Thickness (nm)	Cut-Off Frequency (THz)
Al_2_O_3_–TiO_2_	0.304–1.34	2	29.3
Al_2_O_3_–ZnO	0.304–3.57	2–3	27.0
Al_2_O_3_–HfO_2_	0.304–3.92	2	25.9
TiO_2_–ZnO	1.34–3.57	2–3	10.1
TiO_2_–HfO_2_	1.34–3.92	2–3	9.88
ZnO–HfO_2_	3.57–3.92	2–3	4.88

**Table 5:** Resistance, responsivity, and nonlinear factor values of different combinations of M_1_I_1_I_2_M_2_ diodes extracted from the I-V response. Reprinted with permission from ref. [72]. Copyright 2021 IOP Publishing.

Oxide	Electron Affinity (eV)	Zero-Bias Responsivity (V^−1^)	Dynamic Resistance at Zero-Bias (Ohm)	Nonlinear Factor (at 0.4 V)
Al_2_O_3_/TiO_2_	2/4.05	2.7	580 K	2.3
Al_2_O_3_/ZnO	2/4.2	3.8	400 K	2
Al_2_O_3_/HfO_2_	2/2.14	1.6	380 K	2.1
TiO_2_/ZnO	4.05/4.2	1.8	600 K	2
TiO_2_/HfO_2_	4.05/2.14	3.5	220 K	2
ZnO/HfO_2_	4.2/2.14	7	150 K	2.8
TiO_2_/NiO	4.05/4.9	5	570 K	2.5

**Table 6:** Summary of recent achievements in the field of metal multi-insulator metal (MI^n^M) diode for THz rectennas.

Material	Cut-Off Frequency	*J_ON_*	*Asym*	*NL*	*S* (V^−1^)	Zero-Bias *S* (V^−1^)	Resistance
W/Nb_2_O_5_ (3 nm) /Ta_2_O_5_ (1 nm) /W [59] W/Nb_2_O_5_ (1 nm)/Ta_2_O_5_ (1 nm) /W [59]	Up to 150 THz	- -	- -	- -	11 11	- -	- -
Cr (60 nm)/TiO_2_ (1.5 nm) /Al_2_O_3_ (1.5 nm) /Ti (60 nm) [60] Cr (60 nm)/TiO_2_ (0.75 nm) /Al_2_O_3_ (0.75 nm)/TiO_2_ (0.75 nm)/Al_2_O_3_ (0.75 nm)/Ti (60 nm) [60]	Up to 150 THz	- -	- -	6 7	3 90	- -	- -
Al (60 nm)/Ta_2_O_5_ (3–6 nm)/Al_2_O_3_ (1 nm)/Al (60 nm) [61] Al (60 nm)/Nb_2_O_5_ (3–6 nm)/Al_2_O_3_ (1 nm)/Al (60 nm) [61]	Up to 150 THz	10^2^A/m^2^	18	7.5	9	-	-
Co/Co_3_O_4_ (1.1 nm)/TiO_2_ (1.05 nm)/Ti [62]	Up to 30 THz	10^5^ A/cm^2^	-	-	4.4	2.2	18 KΩ
Ti/TiO_2_ (1 nm)/ZnO (0.5 nm)/Al [63]	Up to 17.4 THz	-	-	-	5.1	1.6	312 Ω
Cr/Cr_2_O_3_ (2 nm)/HfO_2_ (2 nm)/Al_2_O_3_ (2 nm)_/_Cr [64] Cr/Cr_2_O_3_ (2 nm)/Al_2_O_3_ (2 nm)/HfO_2_ (2 nm)/Cr [64]	Up to 30 THz	- -	5 4	4 5	- -	- -	- -
Pt (70 nm)/TiO_2_ (2 nm)/TiO_1.4_ (0.6 nm)/Ti (50 nm) [65]	Up to 30 THz	4.2 × 10^6^ A/m^2^	7.3	-	-	-	-
Cr (100 nm)/Cr_2_O_3_ (3 nm)/Al_2_O_3_ (3 nm)/Ag (100 nm) [66]	Up to 30 THz	3 mA/cm^2^	>280	-	-	-	-
Cr (100 nm)/Al_2_O_3_ (2 nm)/HfO_2_ (2 nm)/Cr [67]	Up to 30 THz	70 µA/cm^2^	9	10	4.8	-	-
ZCAN (ZrCuAlNi 150 nm)/HfO_2_ (5 nm)/Al_2_O_3_ (3 nm)/Al (150 nm) [68]	Up to 30 THz	-	>10	>5	-	-	-
Pt (150 nm)/HfO_2_ (1.5 nm)/TiO_2_ (1.5 nm)/Ti (150 nm) [69,70]	Up to 30 THz	-	10	>5.5	2 × 10^4^	-	0.1 MΩ
Pt (150 nm)/Al_2_O_3_ (1.5 nm)/TiO_2_ (1.5 nm)/Ti (150 nm) [69,70]	Up to 30 THz	-	17	>5.5	2 × 10^4^	-	0.1 MΩ
Ni (150 nm)/NiO (1.5 nm)/ZnO (1.5 nm)/Cr (150 nm) [71]	Up to 30 THz	-	16	-	-	-	-

**Table 7:** Summary of recent achievements in the field of graphene-based geometric diode for THz rectennas.

Diode Configuration	Nanoantenna	Operating Frequency (THz)	Maximum Responsivity (V^−1^)	Zero-Bias Responsivity (V^−1^)	Zero-Bias Resistance (Ω)
Exfoliated monolayer graphene- based arrowhead-shaped diode [83]	metal bowtie 15 nm Cr/40 nm Au	28.3	0.2 for *V_DS_* = 1.5 V	0.18 for *V_DS_* = 0 V	13 K
Exfoliated monolayer graphene- based arrowhead-shaped diode [84]	metal bowtie 15 nm Cr/40 nm Au	Up to 160	0.8 for *V_DS_* = 0.4 V	0.3 for *V_DS_* = 0 V	19 K
Exfoliated monolayer graphene- based arrowhead-shaped diode [85]	metal bowtie 15 nm Cr/40 nm Au	28.3	0.2 for *V_DS_* (V) = 1.4 V	0.12 for *V_DS_* = 0 V	3 K
(CVD) monolayer graphene- based arrowhead-shaped diode [86]	metal bowtie Ti (10 nm)/Au (40 nm)	28.3	0.3 for *V_DS_* (V) = 0.5 V	0.1 for *V_DS_* (V) = 0 V	5 K
Z-Shaped graphene geometric diodes [87]	-	28.3	2.4 for *V_0_* (V) = 0.5 V	-	-

## Data Availability

The data presented in this study are available in the references section.

## Abbreviations

The following abbreviations are used in this manuscript:

IR	Infrared
THz	Terahertz
DC	direct current
EH	energy harvesting
Si	Silicon
S&Q	Shockley–Queisser
EM	electromagnetic
Rectifying antenna	Rectenna
SPPs	Surface Plasmon Polaritons
E	electric field
AC	alternating current
LPF	Low pass filter
η	Overall efficiency
$\eta_{a}$	Antenna collection efficiency
$\eta_{s}$	Losses in the material
$\eta_{c}$	Coupling or matching efficiency
$R_{A}$	Antenna resistance
$R_{D}$	Diode resistance
$C_{D}$	Diode capacitance
ω	Operating frequency
$\eta_{q}$	Quantum efficiency
MIM	Metal-Insulator-Metal
MI^n^M	Metal- multi-insulator-Metal
GD	geometric diode
$f_{c}$	Cut-off frequency
EBL	electron beam lithography
MBE	molecular beam epitaxy
SEM	scanning electron microscopy
MOCVD	metalorganic chemical vapor deposition
PR	Photoresist
ICP	inductively coupled plasma
TMAH	tetramethylammonium hydroxide
PECVD	plasma-enhanced chemical vapor deposition
FOMs	Figure of Merits
Ψ	Work function
*χ*	Electron affinity
*φ*	Barrier height
Δ*φ*	Barrier width
*E_F_*	Fermi levels
DT	direct tunneling
FNT	Fowler–Nordheim Tunneling
I-V	current to voltage
TOV	Turn-on voltage
ZBR	zero-bias resistivity
Sym	Symmetry
*Asym*	Asymmetry
*NL*	Nonlinearity
*S*	Responsivity
RC	Resistance-Capacitance
BG	Band gap
H-BN	hexagonal Boron Nitride
MIG	Metal-insulator-graphene
Gr-h-BN-Gr	graphene/hexagonal boron nitride (h-BN)/graphene heterostructure
ALD	atomic layer deposition
GBG-TD	graphene/h-BN/graphene tunneling diode
CVD	chemical vapor deposition
GrIM	Graphene-Oxide-Metal Diode
AAO	Anodic Aluminum Oxide
AP-CVD	atmospheric pressure chemical vapor deposition
PEALD	Plasma-enhanced atomic layer deposition
PMMA	polymethyl methacrylate
MIBK: IPA	methyl-isobutyl ketone: isopropanol
PVD	Physical Vapor Deposition
MIIM	metal-insulator-insulator-metal
M-I	Metal-interface
I-I	Interface-Interface
I-M	Interface-metal
QW	Quantum Well
MI^2^M	Metal-insulator-insulator-metal
MI^4^M	metal-insulator-insulator-insulator-insulator-metal
IPA	Isopropanol alcohol
MI^3^M	metal-insulator-insulator-insulator-metal
TEM	transmission electron microscope
MFPL	mean-free path length
AFM	atomic force microscopy
*d_n_*	neck width
*d*_s_	Shoulder width
TaN	Tantalum nitride
